# Features of bacterial and fungal communities in the rhizosphere of *Gastrodia elata* cultivated in greenhouse for early harvest

**DOI:** 10.3389/fmicb.2024.1389907

**Published:** 2024-04-24

**Authors:** Nguyen Van Khanh, Swarnalee Dutta, Chang-Su Kim, Yong Hoon Lee

**Affiliations:** ^1^Division of Biotechnology, Jeonbuk National University, Iksan-si, Republic of Korea; ^2^Jinan Medicinal Resource Research Institute, Jeollabukdo Agricultural Research and Extension Services, Jinan-gun, Republic of Korea; ^3^Advanced Institute of Environment and Bioscience, Plant Medical Research Center, and Institute of Bio-industry, Jeonbuk National University, Jeonju-si, Republic of Korea

**Keywords:** *Gastrodia elata*, microbiome, rhizosphere, soil microbes, symbiosis

## Abstract

Symbiotic microbes are essential for developing and growing *Gastrodia elata*, an achlorophyllous orchid of high medicinal value. Recently, the cultivation of *G. elata* in greenhouses has been adopted in Korea to produce mature tubers in a short time. However, no studies have been conducted on the microbial community structure of *G. elata* cultivated in greenhouse environments. Therefore, we analyzed the temporal features of bacterial and fungal communities in the rhizosphere of *G. elata* at the juvenile [JT; 2 months after sowing (MAS)], young (YT; 6 MAS), and mature (MT; 11 MAS) tuber stages using culture-dependent and high-throughput sequencing technology. The richness and diversity of the bacterial and fungal communities decreased with tuber growth of *G. elata.* The symbiotic fungi *Mycena* sp. and *Armillaria* sp. as well as tuber extract inhibited the growth of various soil-inhabiting fungal and bacterial strains, indicating that *G. elata* and its symbiotic fungi play important roles in the selection of rhizosphere microbes. *Mortierella rishikesha* was the most abundant fungal species in the rhizosphere. We also identified the microorganisms potentially beneficial for *G. elata* development during greenhouse cultivation. Tubers and symbiotic fungi actively exert selective pressure on rhizosphere microbes, influencing the diversity and abundance of bacterial and fungal communities as *G. elata* grows. This study is a first report on the temporal microbial community structure of *G. elata* cultivated in greenhouse. The results on the associated microbiome of *G. elata* will help understand their beneficial interactions with *G. elata* and contribute to improvement in cultivation.

## 1 Introduction

*Gastrodia elata,* which is commonly called Cheonma or Tianma in Korea or China, respectively, is highly valued as a medicine and food, primarily because of its various active compounds such as gastrodin, polysaccharides, and organic acids (Lee et al., 2006; Ahn et al., 2007; Chen et al., 2019; Sun et al., 2023). *Gastrodia elata* is mainly produced in East Asian countries such as China, Japan, and South Korea (Yuan et al., 2020). The growth of *G. elata* primarily rely on symbiosis with fungi under natural conditions (Liu et al., 2023). The seeds of *G. elata* do not contain an endosperm, so their germination and development into protocorms only occur when they are provided with adequate nutrients through the digestion of fungal strains of *Mycena* spp. The juvenile tubers of *G. elata* subsequently grow through a symbiotic association with a mycorrhizal fungus *Armillaria mellea* to yield mature tubers (Park and Lee, 2013; Chen et al., 2019; Yu et al., 2022, 2023).

The rhizosphere is a complex and dynamic root area between the plant and microbial community, and the metabolites secreted by microorganisms and plants induce unique properties in each rhizosphere ecosystem (Waldor et al., 2015). Endophytic microorganisms, including fungi, bacteria, and archaea, are inherited through generations or acquired from the environment and colonize plant tissues (Yang et al., 2020). Both endophytic and rhizosphere microbiota contribute to the adaptation of plants to various abiotic and biotic stresses (Bhattacharyya et al., 2015; Bhattacharyya and Lee, 2016; Jansson and Hofmockel, 2020). Moreover, the microbiota inhibit phytopathogens by producing hydrolytic enzymes and antibiotics, nutrient competition, and induction of resistance (Santoyo et al., 2016; Afzal et al., 2019). Hence, understanding rhizosphere and endophytic microbial ecology is key to enhancing plant productivity, stability, and the function of ecosystems (Hueso et al., 2011).

Several studies have characterized the microbiomes of *Gastrodia* species using high-throughput sequencing. Liu et al. (2015) focused on the fungal communities in the surrounding soil and tubers of *G. flavilabella*. Chen et al. (2019) investigated the changes in fungal communities in the surrounding soil and tubers during *G. elata* growth in wild habitat. Yuan et al. (2020) also studied the bacterial and fungal communities in the mycorrhizosphere and rhizosphere soils of cultivated *G. elata*. Zheng et al. (2023) compared the diversity and structure of endophytic bacterial communities in *G. elata* tubers grown in various geographical regions. The effects of different *Armillaria* strains (Yu et al., 2022; Wang et al., 2023) and *Mycena* strains (Yu et al., 2023) on the structure and metabolic function of soil microbial communities, as well as the quality and productivity of *G. elata* have also received recent research attention.

In Korea, the seed (immature) tubers of *G. elata* are sown around April and cultivated until October of the following year, which takes more than a year from sowing to harvesting (Jo et al., 2017; Kim et al., 2017). Recently, the cultivation of *G. elata* in greenhouses has increased to produce mature tubers in a short period. The growth of *G. elata* entirely relies on nutrients provided by symbiotic fungi. Hence, a proposed hypothesis is that the bacterial and fungal communities have played a major role in accelerating the growth of *G. elata* leading to shorten harvest time under greenhouse conditions. However, no studies have investigated the changes in microbial community structure during the growth phases of *G. elata* cultivation in greenhouses.

In this study, we sampled the tubers and surrounding soils at various growth phases to investigate the temporal changes of microbial communities in the rhizosphere soil and tubers of *G. elata* that are cultivated in greenhouses. Bacterial and fungal community structures were analyzed using high-throughput sequencing. In addition, we analyzed the antagonistic activities of tubers and symbiotic fungi to understand the underlying modes of the changes.

## 2 Materials and methods

### 2.1 Cultivation of *Gastrodia elata* in the greenhouse and sample collection

*Gastrodia elata* tubers and their surrounding soil were collected from the greenhouse (E35.76.85 and N127.37.69) at Medicinal Herb Experiment Station in Jeollabuk-do, South Korea. *Gastrodia elata* was sown in early April 2021 using a ternary cropping system and cultivated in a rain-sheltering plastic greenhouse for approximately 12 months until harvest at the end of March 2022. Briefly, three lines of holes were made in the ridges, and *A. mellea* infected chestnut logs (10–12 cm diameter, 30–40 cm length) were placed parallel to the bottom of each hole. After germination of *G. elata* seeds using the symbiotic fungi *Mycena* sp. (Kim et al., 2006), juvenile tubers were sown at regular intervals around the logs. The holes were filled with soil (5–10 cm depth), covered with straw, and soil sprinkled over the surface. Tubers, rhizosphere soil, and bulk soils of *G. elata* were carefully collected at the rooting and juvenile tuber stage (JT; 2 MAS), growing and young tuber stage (YT; 6 MAS), and then at the harvesting and mature tuber stage (MT; 12 MAS; Yuan et al., 2018; Supplementary Figure S1).

Three places (30 × 100 cm) were randomly selected, and intact tubers along with soil 5 cm from the root zone were dug out from the places at each stage and combined to form one biological replicate. The whole rhizocompartment sample was contained in sterile plastic bags and kept at 4°C. Samples were brought to the laboratory and processed within a couple of days to isolate DNA and microbes from the endosphere (root), rhizosphere, and bulk soil samples. The rhizomorphs of *A. mellea* were removed from each sample. Bulk soil easily detached from the tubers was collected and sieved through a 2-mm sieve. Subsequently, the soils adherent to the tubers were detached using sterilized brushes, and taken as rhizosphere soil. The tubers were washed with tap water, then surface-sterilized with ethanol (70%, v/v) for 1 min and sodium hypochlorite solution (3%, v/v) for 3 min, and finally rinsed with sterile distilled water (DW) five times and used as endosphere samples. Successful surface sterilization was confirmed by spreading 100 μL of the final rinsing water on Luria Bertani (LB) agar plates, and bacterial growth was evaluated after incubation at 28°C for 48 h. Three samples from biological bulk soil, rhizosphere soil, and endosphere were analyzed.

### 2.2 Estimation and isolation of culturable bacteria and fungi

The collected soil samples (5 g) were added to 50 mL sterile DW. The suspensions were thoroughly stirred for 5 min, serially diluted, and 100 μL of each dilution was spread on 0.1% tryptic soy agar (TSA) containing 50 μg/mL cycloheximide for total bacterial count, and 10% potato dextrose agar (PDA) containing 50 μg/mL ampicillin and 20 μg/mL rifampicin for total fungal count. The surface-disinfected tuber tissues (1 g) were ground in a sterilized mortar and pestle and suspended in sterile DW (10 mL) to isolate endophytic bacteria and fungi from the tubers. The suspension was serially diluted 10-fold and plated on TSA and PDA media supplemented with 1% tuber extract (TE) of *G. elata*. TSA plates (three replicates for each subsample/dilution combination) were incubated at 28°C for 3 days, and PDA plates were incubated at 25°C for 7 days to enumerate viable bacterial and fungal colonies, respectively. Bacterial colonies showing distinct colors and shapes were randomly picked and transferred to fresh Luria-Bertani (LB) agar medium and stored at −80°C in the LB medium supplemented with 15% glycerol. Fungal isolates showing distinct morphology and color were picked and transferred to test tubes containing PDA slant media and stored at 4°C.

### 2.3 DNA isolation and sequencing

DNA was extracted from each collected sample using the GeneAll Exgene Soil DNA isolation kit (GeneAll, Seoul, South Korea), following the manufacturer’s instructions (Dutta et al., 2022). The quality and quantity of the isolated DNA were analyzed using a BioTek, Epoch Spectrometer (Agilent Technologies, Santa Clara, California, United States) and 1% agarose gel electrophoresis.

Bacterial libraries were generated by amplifying the V3–V4 region of the 16S rRNA gene using the universal primers including 341F and 805R (Dutta et al., 2022). Fungal libraries were produced by amplifying the ITS region using the forward primer ITS3-F and reverse primer ITS4-R (White et al., 1990). These primers appended with the nextera concensus and sequencing adaptor at the 5′ end (Fadrosh et al., 2014). The PCR products were cleaned up and used for further amplification with primers containing Illumina dual indices and sequencing adapter (Dutta et al., 2022). The PCR cycling conditions were a denaturation step at 95°C for 3 min; 25 cycles (1st PCR) and 8 cycles (2nd PCR) of 30 s denaturation at 95°C, 30 s annealing at 55°C, 30 s extension at 72°C; and a 5 min final extension at 72°C. The amount of the PCR product was measured using a Quant-iT PicoGreen dsDNA Assay Kit (Thermo Fisher Scientific, Waltham, Massachusetts, United States), and the quality was assessed using an Agilent Bioanalyzer 2,100 system (Agilent Technologies, Santa Clara, California, United States). Purified amplicon libraries were pooled and sequenced at CJ Bioscience Inc. (South Korea) using an Illumina MiSeq system with MiSeq Reagent Kit v2 (Illumina Inc., San Diego, California, United States). Sequence data were deposited in the GenBank SRA database under BioProject accession numbers PRJNA1074006 and PRJNA1074502 for bacteria and fungi, respectively.

### 2.4 Sequence data processing and analysis

Sequenced data were analyzed using EzBioCloud,^1^ a public pipeline that provides quality control, merging paired-end reads, sequence processing, taxonomic classification, and diversity analysis for operational taxonomic units (OTUs), operated by CJ Bioscience, Inc. (Seoul, South Korea), as described by Dutta et al. (2022). Taxonomic assignments were implemented by searching reference databases using the USEARCH program for the 16S rRNA of bacteria and the UNITE^2^ database for the ITS2 of fungi. Query sequences that are matched to reference sequences in EzBioCloud by ≥97% similarity considered to be at the species level. Lower sequence similarity cutoffs are used for genus or higher taxonomic levels as follows (x = similarity), genus (97 > x ≥ 94.5%), family (94.5 > x ≥ 86.5%), order (86.5 > x ≥ 82%), class (82 > x ≥ 78.5%), phylum (78.5 > x ≥ 75%; Yarza et al., 2014). The suffix (_p, _c, _o, _f, _g, _s) of the taxonomic name is used when the sequence of the database that is most similar to the base sequence to be analyzed does not have a formal taxonomic name (phylum, class, order, family, genus, and species, respectively). Non-cultured species are used to the existence only in the nucleotide sequence data. The suffix “_uc” of the taxonomic name is an abbreviation of unclassified. It is a collection of sequences that can be regarded as new because there is no similar base sequence to the species level in the database. In this case, “_uc” is added to the parent system name to create arbitrary matrix and express the result.

The alpha diversity indices of the bacterial and fungal communities, including ACE, Chao1, Shannon, Simpson, Pielou’s evenness, and phylogenetic diversity indices, as well as rarefaction curves, were calculated. Discrepancies in alpha diversity, such as diversity, number of OTUs, and richness, were analyzed and compared between the samples collected at various growth stages of *G. elata*, and taxonomic compositions were compared from the phylum to the species level. Beta diversity, including principal coordinate analysis (PCoA) and unweighted pair-group method with arithmetic mean (UPGMA) clustering, was analyzed based on the Bray–Curtis dissimilarity index (Bray and Curtis, 1957) at the species level. A heatmap showing the relative abundance of fungi and bacteria at the genus level was analyzed and compared using CL community program v3.43 (CJ Bioscience, Seoul, South Korea; Dutta et al., 2021).

### 2.5 Antagonism assays of *Mycena* sp. and *Armillaria* sp. against fungal strains

The antagonistic activity of *Mycena* sp. Jinan-1 (MJ-1) and *A. mellea* KACC52941 (Am52941) were assayed against soil-inhabiting or plant-pathogenic fungal strains following the methods of Yu et al. (2012) with minor modifications. The fungal strains, such as *Botrytis cinerea*, *Chaetomium novozelandicum, Fusarium graminearum* KACC41040*, Penicillium digitatum*, and *Rhizopus stolonifer* KACC41364 were maintained on PDA slants at 4°C were cultured using fresh PDA plates. Mycelial disk (8 mm) from the active culture of each fungal strain were placed on PDA plate 2.0 cm from the edge of the plate. Similarly, a mycelial disk (8 mm in diameter) of MJ-1, Am52941, or a PDA plug (control) was placed on the opposite side of the plate. The plates were incubated at 25°C for 7 days, and the radial growth of the tested fungi was measured. The radial inhibition rate (RI) was calculated according to the following formula (Nuangmek et al., 2021): RI = (Ro – Rt) /Ro × 100, where Ro represents the radius of tested fungi in the control plates, and Rt represents the radius of tested fungi in treatment plates. The experiment was repeated thrice with three replicates. The KACC strains were originally obtained from Korean Agriculture Culture Collection; *Mycena* sp. Jinan-1 (MJ-1) was donated from the Medicinal Herb Experiment Station; *B. cinerea* and *P. digitatum* were isolated and identified by plant pathology lab of Jeonbuk National University (Dutta and Lee, 2022); *Chaetomium novozelandicum* was isolated and identified from the rhizosphere of *G. elata*.

### 2.6 Inhibition activity of culture filtrate of *Mycena* sp. and *Armillaria* sp.

Culture filtrates (CFs) from MJ-1 and Am52941 cells were obtained using method described by Yu et al. (2012). Briefly, the actively growing mycelium plug from freshly cultured plate was inoculated into 100 mL of potato dextrose broth (PDB) in a 250 mL Erlenmeyer flask and incubated at 27°C for 4 weeks with slow shaking. The culture broth was collected by filtering through Whatman No. 4 filter paper and then centrifuged at 10,000 rpm for 20 min to remove cellular debris, followed by filtering the supernatant through a 0.22 μm filter. The CFs were stored at −20°C until further use. The antagonistic activity of CF against the growth of fungal strains, such as *B. cinerea*, *C. novozelandicum, F. graminearum, P. digitatum*, and *R. stolonifer*, was assessed by mixing a gradual two-fold CF dilution with autoclaved, molten PDA at 45°C to eventual concentrations of 50%, 25%, 12.5%, and 6.25%. Fresh PDA was used as a control. Mycelial disks (8 mm in diameter) were removed from the fringe of a 5- to 7-day-old colony of each fungal strain grown on PDA and placed on the center of the PDA plates. The plates were maintained at 25°C in darkness, and the diameters of fungal colonies were measured after 10 days of growth. The percentage of inhibition was calculated using the following formula (Kong et al., 2018): [(D_o_ – D_t_) /D_o_] × 100, where D_o_ represents the average colony diameter of the control, and D_t_ represents the average colony diameter of the treatment. All treatments were effectuated independently thrice in triplicate.

The antagonistic activity of the CF against Gram-positive (*Bacillus subtilis* KACC10854, *B. velezensis*, and *Cytobacillus firmus* JBRS159) and Gram-negative (*Agrobacterium tumefaciens*, *Pseudomonas putida* JBC17, and *Variovorax paradoxus* JBCE486) bacteria was assessed by spreading a 100 μL of cell suspension of each strain on LB agar plates amended with various concentrations (6.25%, 12.5%, 25%, 50%) of CF of MJ-1 or Am52941. The number and size of the bacterial colonies were determined after incubation at 28°C for 48 h. The experiment was replicated thrice with three replicates. The KACC strains were originally obtained from Korean Agriculture Culture Collection; Other strains were stock culture of plant pathology lab of Jeanbuk National University (Dutta et al., 2022; Dutta and Lee, 2022; Kang et al., 2023).

### 2.7 Inhibition activity of tuber extract

Mature tubers of *G. elata* were peeled, surface-sterilized with ethanol and sodium hypochlorite, and washed with sterile DW as described above. Tuber tissues (200 g) were sliced using a sterilized knife and ground with a mortar and pestle. The homogenates were resuspended in sterile DW (200 mL) and shaken for 2 h at 200 rpm. The slurry was filtered through four layers of cheesecloth, and then the suspension was filtered sequentially through Whatman filter paper and a sterile syringe filter (0.2 μm pore size) to create tuber extract (TE, 100%). The LB agar and PDA medium containing TE at various concentrations (6.25%, 12.5%, 25%, and 50%) were used to evaluate the inhibitory activity of TE against bacterial and fungal strains, respectively, as described above.

### 2.8 Statistical analysis

All the experiments were performed using a completely randomized design. The statistical significance of OTU numbers, alpha diversity of microbial communities, and relative abundance at taxonomic ranks among different growth stages of *G. elata*, as well as the size of the tested fungal colonies in the antagonism experiments, was analyzed by one-way analysis of variance (ANOVA) and Tukey’s test at the *p* < 0.05 level of significance using Minitab version 16.2.0 software (Bower, 2000).

## 3 Results

### 3.1 Temporal changes of culturable bacterial and fungal populations

Cultural management and soil properties for crop cultivation induce changes in the quality and quantity of microbiota. In this study, *G. elata* tubers and surrounding soils following the growth stages were used to investigate the characteristics of the microbial communities associated with the various compartments of *G. elata* cultivated in greenhouses. The soils were loosely attached to the tubers, making it difficult to distinguish between the rhizosphere and the bulk soil around the tubers (Supplementary Figure S1). There were no significant differences in the total culturable bacterial and fungal population densities in the bulk and rhizosphere soils of *G. elata* plants (Supplementary Figure S2). Almost no bacterial and fungal isolates were obtained from the tubers, except for a few strains from the skin of the tubers. Our findings indicated no notable distinctions in the quantities of culturable bacteria and fungi between bulk and rhizosphere soils, and no culturable microbes were present as endophytes.

### 3.2 16S rDNA and ITS2 sequencing data and alteration of alpha diversity

We analyzed the bacterial and fungal community structures of *G. elata* at various growth stages. High-throughput sequencing generated 570,245 (median: 63,360) bacterial 16S rDNA reads and 617,010 (median: 68,557) fungal ITS region reads. After quality trimming, merging, and removal of chimeric reads, a total of 451,059 and 355,536 reads with median read lengths of 416.74 and 339.55 for bacteria and fungi, respectively, were obtained. The rarefaction curve at 3% dissimilarity in rhizosphere showed an average of 4,713, 3,201, and 2,683 OTUs for bacteria and an average of 696, 573, and 484 OTUs for fungi in JT, YT, and MT stages, respectively (Supplementary Figure S3; Supplementary Tables S1, S2), indicating that the sequencing depth adequately covered detectable species in rhizosphere soil samples. The Good’s coverage analysis revealed more than 98% and 99% of the bacterial and fungal taxonomic richness, respectively, which were covered by sequencing endeavors in all the samples of growth stages of *G. elata*, indicating that they represented the actual rhizosphere bacterial and fungal populations in the microbial community of each sample (Supplementary Tables S1, S2).

The highest alpha diversity (Shannon, Chao1, ACE, Simpson, Jackknife, OTUs, NPShannon, and phylogenetic diversity) of the rhizosphere bacterial and fungal communities was observed in the JT samples, and the indices were not significantly different between the YT and MT samples. Pielou’s evenness index showed that the microbial communities were evenly distributed in the JT samples (Supplementary Tables S1, S2). The indices indicated that the richness and diversity of bacterial and fungal communities in YT and MT were lower than in JT.

### 3.3 Comparison of bacterial and fungal community structures of *Gastrodia elata* using beta diversity analysis

**Figure 1:**
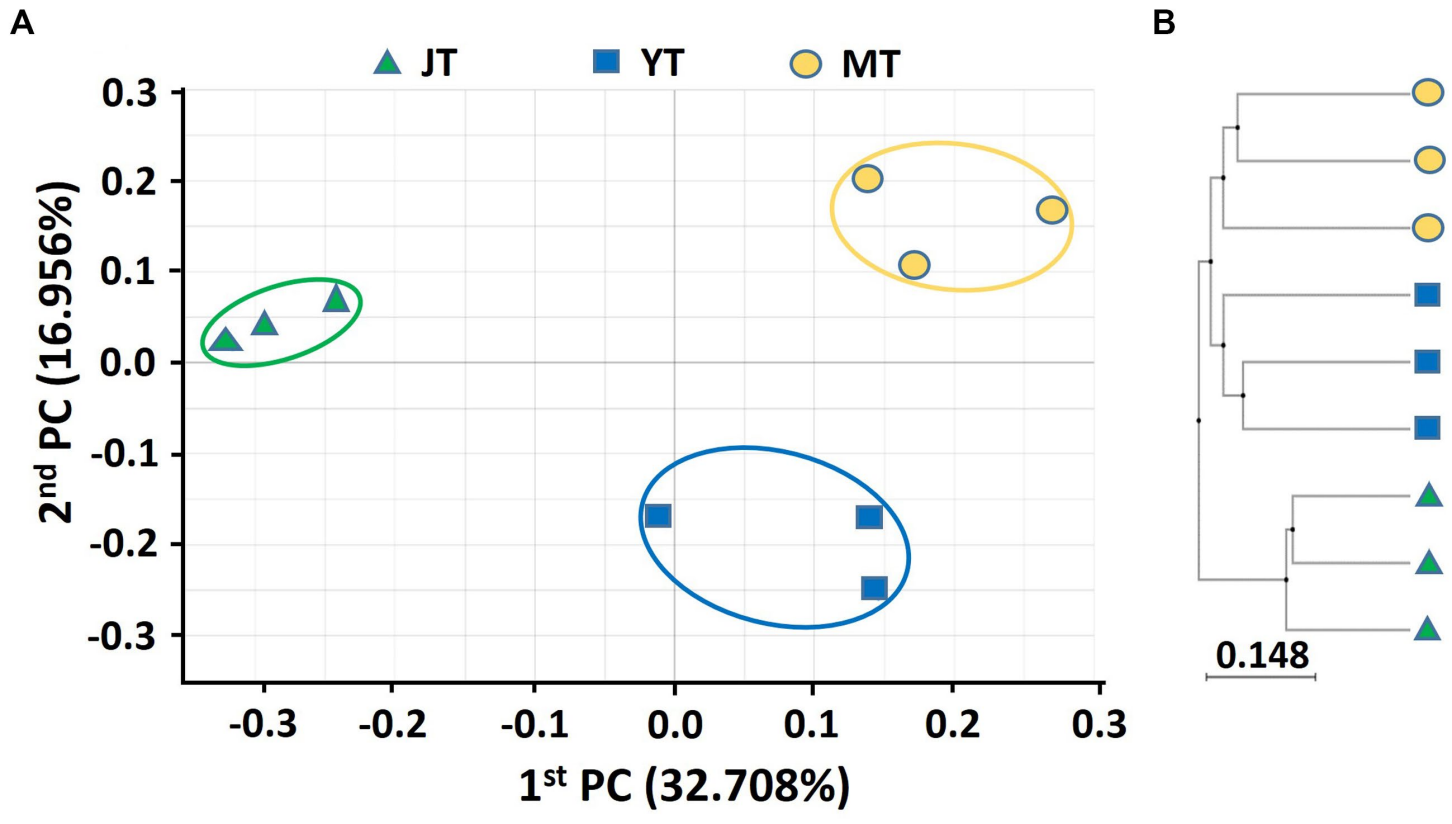
Principal coordinates analysis (PCoA) and unweighted pair-group method with arithmetic mean (UPGMA) clustering of bacterial microbiota. **(A)** PCoA and **(B)** UPGMA clustering were analyzed using Bray–Curtis dissimilarity. Bacterial communities from the rhizosphere of *Gastrodia elata* at the rooting and juvenile tuber (JT), growing and young tuber (YT), and harvesting and mature tuber (MT) stages in the greenhouse were compared using the relative abundance of all operational taxonomic units (OTUs).

A PCoA based on Bray–Curtis analysis was performed, and a UPGMA clustering tree was built using 16S rDNA and ITS sequences to compare the bacterial and fungal community structures in rhizosphere soils at various growth stages of *G. elata*. The PCoA analysis of bacterial communities showed a coefficient of variation of 49.67%, indicating that they could characterize the bacterial community composition in rhizosphere. The beta diversity indices of the PCoA revealed that the bacterial taxonomic structure and composition at each growth stage were distinctly clustered, indicating changes in the bacterial community structure over time. The JT stage samples were closely grouped in comparison to the YT and MT stages, indicating that the bacterial community composition in the YT and MT stages was more diverse between the locations (Figure 1A). The UPGMA clustering also showed that the species-level bacterial community structures of the JT, YT, and MT stages clustered differently (Figure 1B).

**Figure 2:**
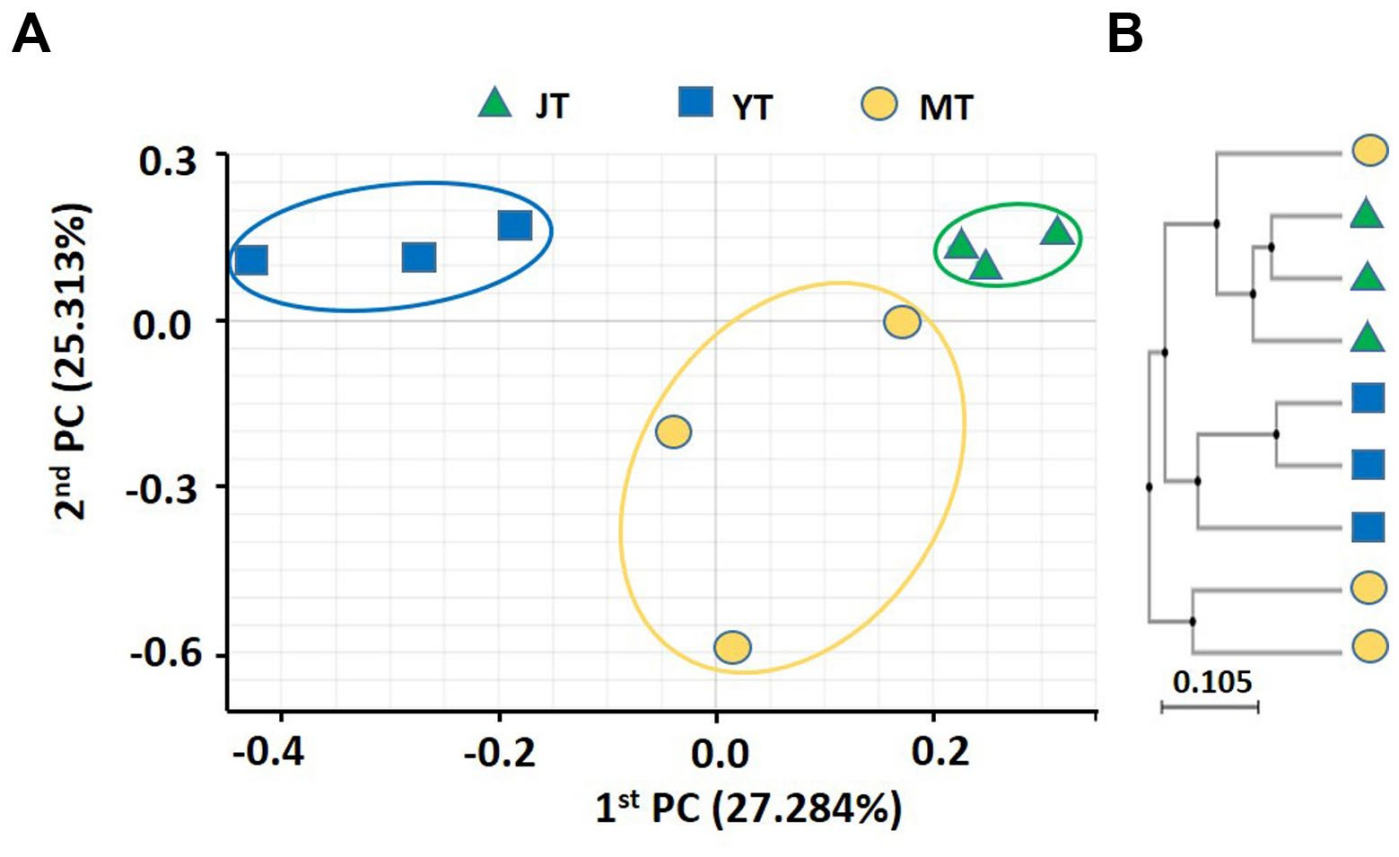
Principal coordinates analysis (PcoA) and unweighted pair-group method with arithmetic mean (UPGMA) clustering of fungal microbiota. **(A)** PCoA and **(B)** UPGMA clustering were analyzed using Bray–Curtis dissimilarity. Fungal communities from the rhizosphere of *Gastrodia elata* at the juvenile tuber (JT), young tuber (YT), and mature tuber (MT) stages in the greenhouse were compared using the relative abundance of all operational taxonomic units (OTUs).

The coefficients of variation of PCoA for fungal community in *G. elata* rhizosphere were 27.28% and 25.31% for PC1 and PC2, respectively. The fungal communities in JT, YT, and MT were distinctly separated, indicating that their fungal community composition changed during the growth stages. The fungal community at the JT stage was more closely grouped than that at the YT and MT stages, indicating less variation in the fungal community composition at the JT stage than at the YT and MT stages (Figure 2A). The UPGMA clustering showed that the species-level fungal community structures of MT were widely scattered among the locations (Figure 2B). The fungal communities were distinctly separated at JT and YT. However, there were similarities between the fungal communities at MT with that of JT and YT, respectively. Taken together, the structures of bacterial and fungal communities in the rhizosphere of *G. elata* showed distinct clusters over time. These results indicated a unique bacterial and fungal composition at each growth stage of *G. elata*.

### 3.4 Temporal changes of bacterial communities

**Figure 3:**
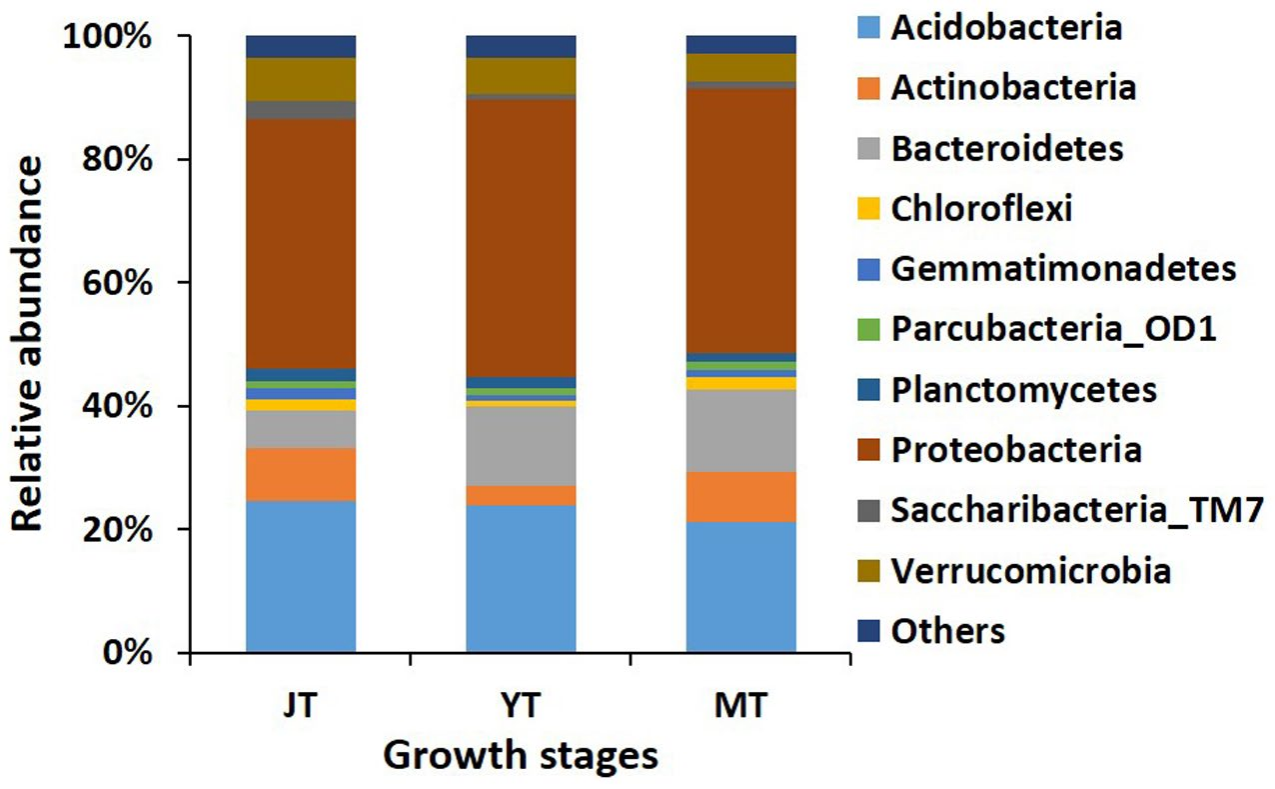
Comparison of relative abundance of bacterial composition at phylum level. All operational taxonomic units (OTUs) of bacterial phyla with relative abundances higher than 1% in the rhizosphere of *Gastrodia elata* at juvenile tuber (JT), young tuber (YT), and mature tuber (MT) stages in the greenhouse were compared. Phyla with relative abundances below 1% were categorized as “Others”.

We compared the bacterial taxonomic profiles of the rhizosphere soils collected at various growth stages of *G. elata*. A total of 44, 44, and 37 bacterial phyla were detected in the JT, YT, and MT stages, respectively. The dominant bacterial phyla in the rhizosphere of *G. elata* were consistently abundant throughout the growth stages of *G. elata*. Proteobacteria was the most abundant phylum in all stages (40.61%–45.17%), followed by Acidobacteria (21.15%–24.61%), and Bacteroidetes (6.098%–13.21%; Figure 3; Supplementary Table S3).

**Figure 4:**
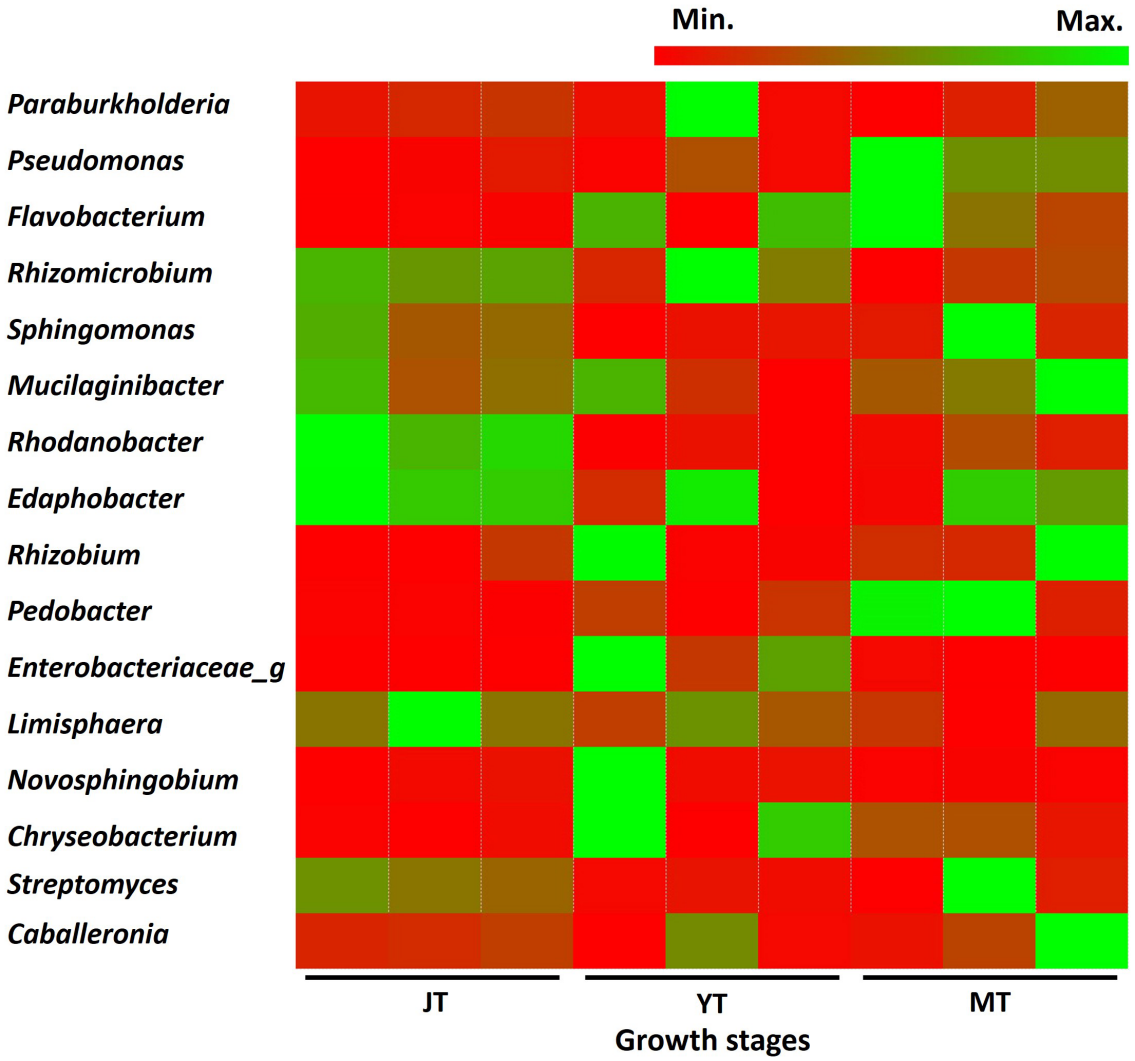
Comparison of relative abundance of bacteria at genus level. The bacterial genera exhibiting relative abundances exceeding 1% in rhizosphere of *Gastrodia elata* at the juvenile tuber (JT), young tuber (YT), and mature tuber (MT) stages in the greenhouse were compared.

In total, 616, 550, and 476 genera with more than 0.01% relative abundance were identified at the JT, YT, and MT stages, respectively. Among these, 353 genera were consistently detected in all growth stages of *G. elata,* comprising 57%, 64%, and 74% of the total population at the JT, YT, and MT stages, respectively. The numbers of unique bacterial genera at the JT, YT, and MT stages were 139, 89, and 49, respectively (Supplementary Figure S4). The genera such as *Paraburkholderia* (3.38%–5.97%), *Rhizomicrobium* (2.35%–4.44%), *Mucilaginibacter* (1.72%–3.33%), *Sphingomonas* (1.73%–3.99%), and *Edaphobacter* (1.58%–2.98%) were constantly detected with high abundance. The average relative abundances of *Pseudomonas* and *Rahnella* increased with the growth of *G. elata,* whereas *Solibacter, Acidibacter, Bradyrhizobium,* and *Rhodanobacter* decreased (Figure 4; Supplementary Table S4). These results indicated that the number of bacterial genera gradually decreased with the growth of *G. elata,* whereas some strains remained constant throughout the growth period.

At the bacterial species level, *Edaphobacter dinghuensis*, *Sphingomonas lutea*, and *Bradyrhizobium japonicum* were highly abundant at all growth stages of *G. elata*. The relative abundance of *Rhodanobacter glycinis* at the JT stage was higher than that at the YT and MT stages. In contrast, increases in relative abundance were recorded for *Pseudomonas* species such as *Pseudomonas*_uc and *Pseudomonas putida* group (Supplementary Figure S5). Overall, the results indicated that the bacterial community in the rhizosphere changed as the tubers grew, suggesting an active selection of bacterial communities by *G. elata* tubers.

### 3.5 Temporal changes of fungal communities

**Figure 5:**
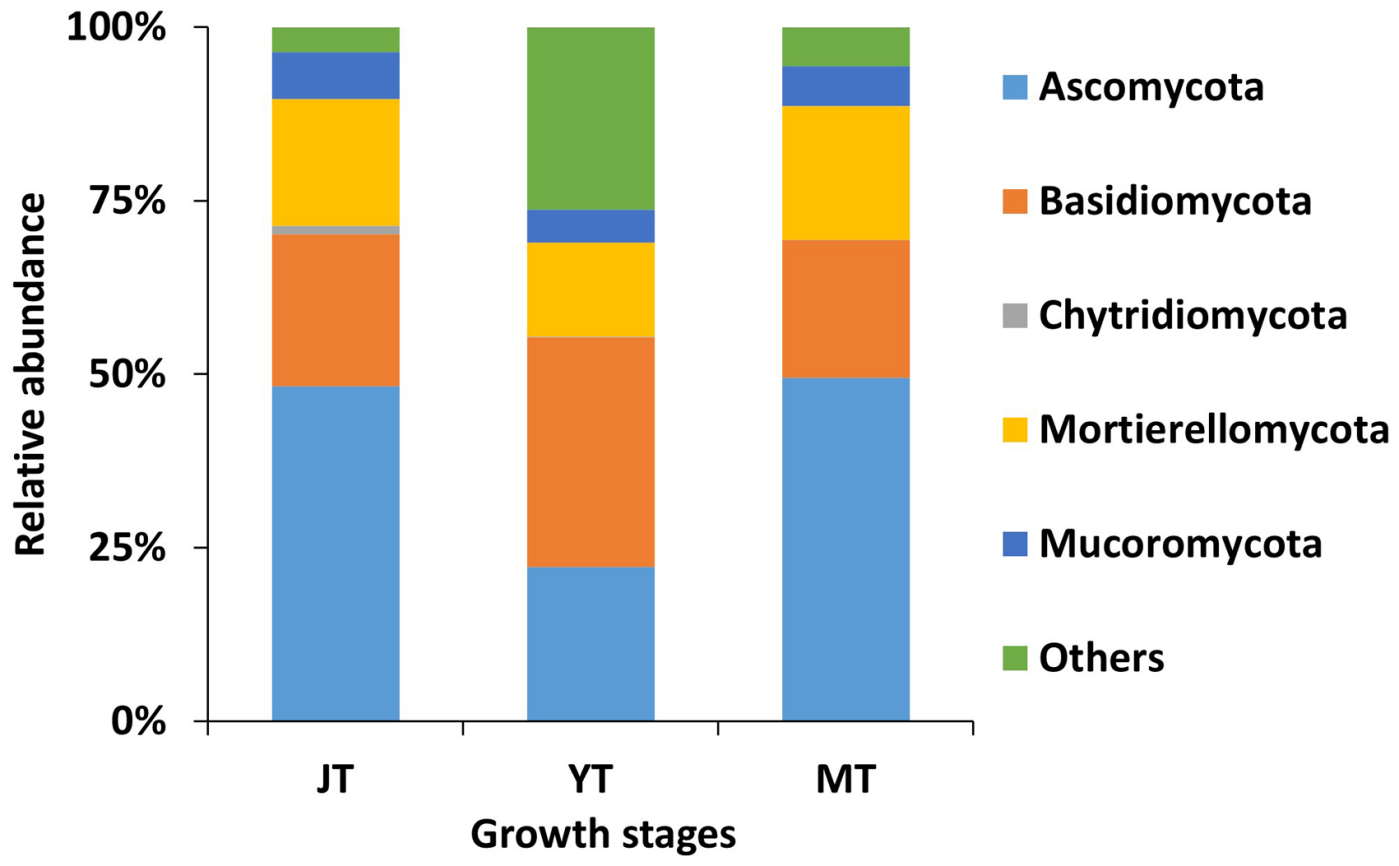
Comparison of relative abundance of fungal composition at phylum level. All operational taxonomic units (OTUs) of fungal phyla with relative abundances higher than 1% in the rhizosphere of *Gastrodia elata* at the juvenile tuber (JT), young tuber (YT), and mature tuber (MT) stages in the greenhouse were compared. Phyla with relative abundances less than 1% were categorized as “Others”.

A total of 14, 12, and 12 fungal phyla were detected in the JT, YT, and MT stages, respectively. The dominant fungal phyla across the growth stages of *G. elata* were Ascomycota (22.23%–49.48%), Basidiomycota (19.90%–33.10%), and Mortierellomycota (13.59%–19.30%; Figure 5; Supplementary Table S5).

**Figure 6:**
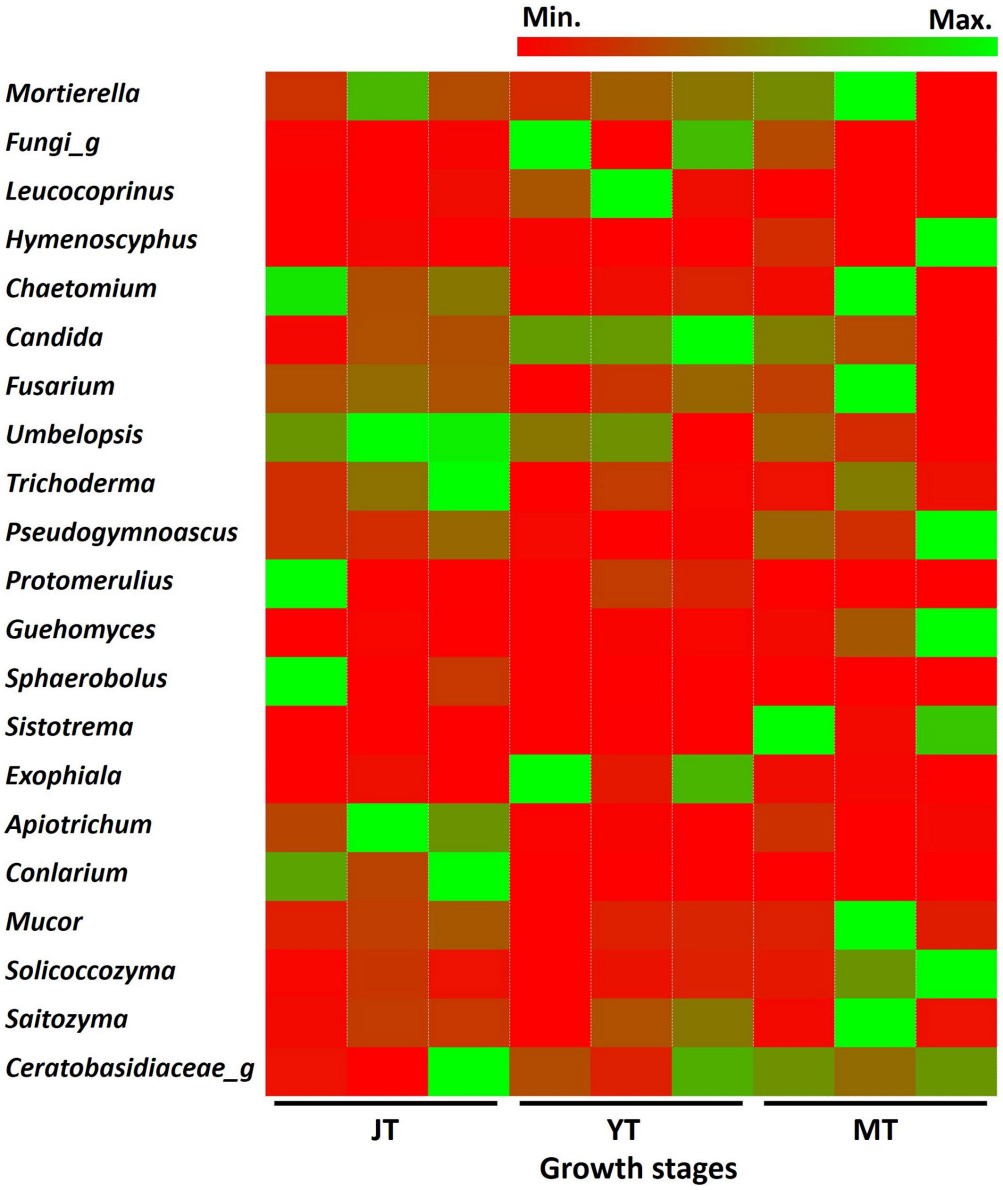
Comparison of relative abundance of fungi at genus level. The fungal genera with relative abundance above 1% in the rhizosphere of *Gastrodia elata* at juvenile (JT), young tuber (YT), and mature tuber (MT) stages were compared.

As the tubers grew, the number of fungal genera in JT, YT, and MT gradually decreased to 335, 266, and 265, respectively. Among the detected fungal genera, 168 were consistently present at all growth stages. A total of 209, 203, and 184 genera were shared between the JT and YT, JT and MT, and YT and MT stages, respectively. By contrast, 91, 41, and 46 genera were unique to the JT, YT, and MT stages, respectively (Supplementary Figure S6). The abundance of fungal genera such as *Capnodiales_g, Conlarium, Oidiodendron, Sordariomycetes_g, Apiotrichum, Mortierellaceae_g,* and *Umbelopsis* decreased as *G elata* tubers grew. The relative abundances of *Geomyces*, *Guehomyces*, *Hymenoscyphus*, *Leptodontidium*, *Mucor*, *Pseudogymnoascus*, *Sistotrema,* and *Solicoccozyma* increased with the growth of *G. elata*. The fungal genera such as *Mortierella* (13.17%–19.09%), *Chaetomium* (1.43%–9.03%), *Trichoderma* (1.04%–3.30%), *Fusarium* (1.90%–2.79%), *Saitozyma* (1.03%–1.44%) were highly abundant throughout the growth of *G. elata*, which indicate possible involvement of these genera in the growth of *G. elata* (Figure 6; Supplementary Table S6). The *Mycena* genus, which plays an important role in the germination of *G. elata* seeds (Xu and Guo, 1989; Chen et al., 2019) was detected with low abundance (none at JT stage and 0.013% and 0.008% at YT and MT stages, respectively).

*Mortierella rishikesha* was the most abundant fungal species in the rhizosphere soils of JT, YT and MT stages (11.1%, 9.7%, and 14.5%, respectively), followed by *Candida subhashii* (1.9%, 4.1%, and 2.1%, respectively). Among the fungal species with relative abundance above 1.0%, *Mortierella rishikesha, Candida subhashii*, *Fusarium oxysporum*, *Saitozyma podzolica*, and *Umbelopsis isabellina* were highly abundant across all growth stages. The abundance of *Oidiodendron rhodogenum*, *Apiotrichum xylopini*, *Capnodiales* sp._GU721261, *Mortierellaceae* sp._HQ445985, *Conlarium* sp._JX489782, and *Trichoderma viride* decreased with the growth of *G. elata* (Supplementary Figure S7).

### 3.6 Antagonism of *Mycena* sp. and *Armillaria* sp. against fungi

**Figure 7:**
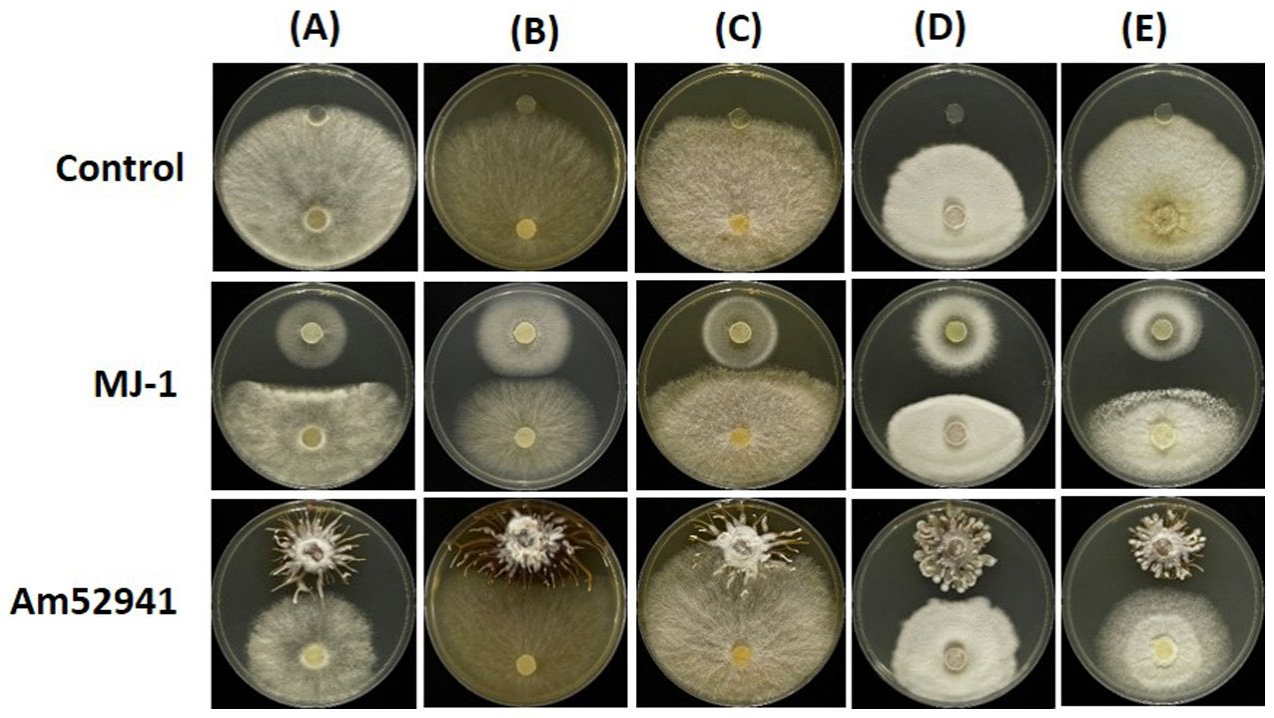
Antifungal activity of *Mycena* sp. and *Armillaria mellea* against fungal strains. An agar plug (8 mm diameter) from the margin of a 5-to 7-day-old culture of **(A)** *Botrytis cinerea*; **(B)** *Chaetomium novozelandicum;* **(C)** *Fusarium graminearum;* **(D)** *Penicillium digitatum*; **(E)** *Rhizopus stolonifer* were placed on PDA plates at the distance of 5 cm away from agar plug of *Mycena* sp. Jinan-1 (MJ-1) or *Armillaria mellea* KACC52941 (Am52941). The agar plugs that were not inoculated with *Mycena* sp. Jinan-1 or *Armillaria mellea* KACC52941 were used as control. The photos were taken 7 days after incubation at 25°C.

The proliferation of symbiotic fungi around seeds and tubers can influence the bacterial and fungal communities in the rhizosphere. The results showed the symbiotic fungus MJ-1 suppressed the growth of soil-inhabiting fungi such as *B. cinerea* (55.4% in radial inhibition rate), *C. novozelandicum* (48.9%)*, F. graminearum* (39.4%), *P. digitatum* (46.1%) and *R. stolonifer* (54.7%). Another symbiont Am52941 inhibited the mycelial growth of *B. cinerea* (34.9%)*, C. novozelandicum* (16.7%), *P. digitatum* (23.4%), and *R. stolonifer* (39.5%), but did not inhibit the growth of *F. graminearum* (Figure 7; Supplementary Table S7).

### 3.7 Antifungal and antibacterial activities of CF of *Mycena* sp. and *Armillaria* sp.

The CFs of MJ-1 and Am52941 also exhibited different antagonistic activities against different fungal and bacterial strains. In which, the CF of MJ-1 significantly suppressed mycelial growth of all tested fungal strains, including *B. cinerea*, *C. novozelandicum*, *F. graminearum*, *P. digitatum,* and *R. stolonifer*. The CF of Am52941 inhibited the growth of *B. cinerea, C. novozelandicum* and *R. stolonifera*, but did not inhibit the growth of *F. graminearum* and *P. digitatum* (Table 1; Supplementary Figure S8).

**Table 1:** Antagonism of culture filtrates of *Mycena* sp. Jinan-1 and *Armillaria mellea* KACC52941 against mycelial growth of fungi.

Fungal strain	Mycelial diameter (mm)								
	Control	*Mycena* sp. filtrate				*Armillaria* sp. filtrate			
		6.25%^*^	12.5%	25%	50%	6.25%	12.5%	25%	50%
*Botrytis cinerea*	65.0^a^	46.5^b^ (28.5)^**^	39.3^bc^ (39.6)	33.8^cd^ (48.1)	27.8^d^ (57.3)	65.3^a^ (NI)	68.0^a^ (NI)	67.0^a^ (NI)	61.5^a^ (5.4)
*Chaetomium novozelandicum*	65.6^a^	54.5^b^ (16.9)	52.3^bc^ (20.4)	47.3^c^ (28.0)	28.3^d^ (56.9)	56.3^b^ (14.3)	54.5^b^ (16.9)	53.0^b^ (19.2)	52.5^bc^ (20.0)
*Fusarium graminearum*	46.7^b^	38.5^c^ (17.5)	35.3^cd^ (24.5)	30.5^d^ (34.6)	30.0^d^ (35.7)	48.8^ab^ (NI)	49.8^ab^ (NI)	49.3^ab^ (NI)	54.0^a^ (NI)
*Penicillium digitatum*	60.7^a^	50.0^b^ (17.6)	41.0^c^ (32.4)	32.5^d^ (46.4)	21.3^e^ (65.0)	61.5^a^ (NI)	66.0^a^ (NI)	64.5^a^ (NI)	68.0^a^ (NI)
*Rhizopus stolonifer*	57.8^a^	46.0^cd^ (20.5)	44.6^de^ (22.8)	41.8^e^ (27.8)	38.0^f^ (34.3)	50.8^b^ (12.3)	52.0^b^ (10.1)	48.5^bc^ (16.1)	46.5^cd^ (19.6)

^*^Concentrations of culture filtrates added to PDA medium. ^**^Inhibition rate (%), NI, no inhibition. The data represent the mean mycelial diameter of three replicates, and significant differences are indicated by different letters in the same row (ANOVA, *p* < 0.05).

The CF of MJ-1 suppressed the growth of *B. velezensis* and *B. subtilis* at 25% concentration, and that of *C. firmus* was significantly inhibited at a concentration of 50%. The growth of Gram-negative *P. putida* and *A. tumefacien* was not inhibited by CF of MJ-1. However, the survival of *V. paradoxus* was significantly suppressed by the CF addition at 6.25% concentration. The CF of Am52941 inhibited the growth of Gram-positive *B. subtilis* at 25% concentration and Gram-negative *V. paradoxus* at 12.5% concentration (Table 2; Supplementary Figure S9). Overall, the results showed that both symbiotic fungi of *G. elata* had different effects on different bacterial and fungal strains, indicating that they play a role in selecting the composition of the rhizosphere microbial community of *G. elata*.

**Table 2:** Effect of culture filtrate of *Mycena* sp. Jinan-1 and *Armillaria mellea* KACC52941 on bacterial survival.

Bacterial strain	Survival rate (%) compared to that of the control							
	*Mycena* sp. filtrates				*A. mellea* filtrates			
	6.25%^*^	12.5%	25%	50%	6.25%	12.5%	25%	50%
*Bacillus subtilis*	100	96	84	82	100	100	94	87
*Bacillus velezensis*	92	93	90	85	100	100	100	100
*Cytobacillus firmus*	100	100	100	22	100	100	100	100
*Agrobacterium tumefaciens*	100	100	100	100	100	100	100	100
*Pseudomonas putida*	100	100	100	100	100	100	100	100
*Variovorax paradoxus*	75	18	14	9	100	31	25	17

^*^Concentrations of culture filtrates added to LB agar.

### 3.8 Antagonistic activity of TE against fungi and bacteria

No fungal or bacterial strains were detected in the tubers in culture-dependent assays for fungal and bacterial populations. In addition, the diversity and relative abundance of microbes decreased as *G. elata* grew. The tuber extracts were assessed for effect on fungal and bacterial growth. The addition of 25% TE inhibited the growth of *B. cinerea* (13.1%), *C. novozelandicum* (7.8%), and *P. digitatum* (22.1%), and the inhibition zone increased with increasing concentrations (50%) of TE. Intriguingly, the growth of *F. graminearum* was significantly increased by the addition of TE (from 29.1% to 52.0%) without any inhibition, and the growth of *R. stolonifer* was inhibited at low concentration (6.25% to 12.5%) but was significantly increased by adding 50% TE (Table 3; Supplementary Figure S10).

**Table 3:** Antagonism of tuber extract of *Gastrodia elata* against mycelial growth of fungi.

Fungal strain	Mycelial diameter (mm)				
	Control (mm)	Concentration of tuber extract			
		6.25%	12.5%	25%	50%
*Botrytis cinerea*	65.0^a^	59.8^b^ (8.1)^*^	57.5^bc^ (11.5)	56.5^c^ (13.1)	45.5^d^ (30.0)
*Chaetomium novozelandicum*	65.6^a^	64.0^ab^ (NI)	62.3^ab^ (5.0)	60.5^b^ (7.8)	43.4^c^ (33.8)
*Fusarium graminearum*	46.7^d^	65.0^b^ (NI)	71.0^a^ (NI)	60.3^c^ (NI)	66.8^b^ (NI)
*Penicillium digitatum*	60.7^a^	63.8^a^ (NI)	68.3^a^ (NI)	47.3^b^ (22.1)	36.4^c^ (40.0)
*Rhizopus stolonifer*	57.8^b^	47.5^c^ (17.9)	46.5^c^ (19.6)	54.0^b^ (6.63)	71.5^a^ (NI)

^*^Inhibition rate (%); NI, no inhibition. The data represent the mean mycelial diameter of three replicates, and significant differences are indicated by different letters in the same row (ANOVA, *p* < 0.05).

The addition of 12.5% TE significantly reduced the survival of the Gram-positive bacteria *B. subtilis* and *B. velezensis*. *Cytobacillus firmus* was completely inhibited by the addition of 25% TE to the medium. The survival of Gram-negative *A. tumefaciens* decreased with the addition of 6.25% TE and decreased further as the TE concentration increased. However, the survival of *P. putida* was not affected by adding TE and that of *V. paradoxus* was inhibited at a concentration of 50% TE (Table 4; Supplementary Figure S11). The differences in the antimicrobial activity of TE against bacterial and fungal species indicated that *G. elata* tubers greatly contribute to the selection of components in the rhizosphere microbial community, leading to a reduction in the diversity of bacterial and fungal communities with the growth of *G. elata*. The colony sizes of most tested bacterial strains decreased with increasing of TE concentrations. However, the colony sizes of *A. tumefaciens, B. velezensis*, and *V. paradoxus* were increased by the addition of low concentrations (6.25%, 12.5%, and 25%) of TE, and colony colors were changed (Supplementary Figure S11). This indicated that tubers also influence physiology and growth.

**Table 4:** Effect of tuber extract of *Gastrodia elata* on bacterial survival.

Bacterial strain	Survival rate (%) compared to that of control			
	6.25%^*^	12.5%	25%	50%
*Bacillus subtilis*	100	79	63	48
*Bacillus velezensis*	93	62	49	31
*Cytobacillus firmus*	100	34	0	0
*Agrobacterium tumefaciens*	85	68	61	35
*Pseudomonas putida*	100	100	100	100
*Variovorax paradoxus*	100	100	100	13

*Concentration of tuber extract added to LB agar.

## 4 Discussion

Cultivation of *G. elata* in greenhouses has been adopted recently to reduce the time needed to produce matured tubers. However, no studies have investigated the changes in microbial community structure during the growth of *G. elata* cultivated in a greenhouse. Therefore, we analyzed the bacterial and fungal communities at various growth phases of *G. elata* growing in a greenhouse using culture-dependent and -independent technologies and the underlying reasons were explored. Our results showed that the richness and diversity of the bacterial and fungal communities in the rhizosphere *G. elata* decreased with the growth. Dutta et al. (2022) indicated that rhizosphere microbes are selected and shaped by plant species through the secretion of compounds that promote or inhibit microorganisms. Chen et al. (2019) observed a decrease in the richness and diversity of fungi in the rhizosphere during *G. elata* growth in wild habitats. Liu et al. (2022) showed that the bacterial community diversity in cultivated soil of *G. elata* was significantly lower than that in uncultivated soil. These findings suggested that *G. elata* regulates the structure of rhizosphere microbial communities.

In this study, we found that Proteobacteria was the predominant phylum in the rhizosphere of *G. elata*, followed by Acidobacteria, which corresponds to the bacterial communities in the cropping soil of *G. elata* (Yuan et al., 2020) and in the surrounding soil of *G. elata* protocorms (Yu et al., 2023). Yu et al. (2023) reported that Proteobacteria showed a positive relationship with soil ammonium nitrogen content in the soil surrounding *G. elata.* In this study, *Paraburkholderia*, *Rhizomicrobium*, *Sphingomonas*, and *Rhizobium* that belong to Proteobacteria, *Edaphobacter* belonging to Acidobacteria, and *Mucilaginibacter* belonging to Bacteroidetes maintained high relative abundances (>1%) during the growth of *G. elata* under greenhouse. In addition, the relative abundances of Proteobacteria such as *Pseudomonas* and *Rahnella,* and Bacteroidetes tended to increase with the growth of *G. elata*. *Pseudomonas* species have been identified as an endosymbiotic group in terrestrial orchids (Wilkinson et al., 1994) and epiphytic orchids (Tsavkelova et al., 2016), and play crucial roles as biocontrol agents that can enhance the growth and health of plants (Weller, 2007; Höfte and Altier, 2010). The relative abundance of *Rahnella* was higher in *G. elata*-cultivated soil samples than in uncultivated soil samples, and the co-inoculation of *G. elata* and *A. gallica* with *Rahnella* sp. HPDA25 promotes the growth of *G. elata* and *A. gallica* (Liu et al., 2022). The resistance of *Pseudomonas* strain against TE of *G. elata,* MJ-1, and Am52941 presumably affected the increase of the abundance, which consequently contributing to the abundance of the genera. The results indicated that beneficial bacteria were selected and maintained at a high abundance during the growth of *G. elata* under greenhouse conditions.

*G. elata* interacts with various fungi, beyond *Mycena* species, during its growth. *Ascomycota*, *Basidiomycota, Mortierellomycota,* and *Zycomycota* are the predominant fungal phyla in the rhizosphere and tubers of *G. elata* growing in the wild (Chen et al., 2019) and farming systems (Yuan et al., 2020). In the present study, the fungal community structure in the rhizosphere of *G. elata* changed during the growth phases. *Ascomycota*, *Basidiomycota*, and *Mortierellomycota* were the dominant fungal phyla during the growth of *G. elata*. *Chaetomium*, *Mortierella*, *Trichoderma*, *Fusarium,* and *Saitozyma* were the most abundant genera throughout the growth period of *G. elata* in this study. *Chaetomium* species have shown the potential for development as biofertilizers by producing cellulase to decay wood and degrade cellulose materials, antibiotics, and ergosterol to enhance soil fertility (Song and Soytong, 2017). *Trichoderma* species are popular biocontrol agents as well as significant growth-promoting orchid plants (Gutiarrez-Miceli et al., 2008; Yuan et al., 2009). *Saitozyma* degrades plant cellulose (Wang et al., 2022). Therefore, our data show that litter-or wood-decaying fungi, such as *Mortierella*, *Chaetomium*, *Trichoderma*, *Fusarium*, and *Saitozyma* species, may provide carbon and nutrients to the symbiotic fungi of *Gastrodia* species.

*Mortierella rishikesha* maintained highest relative abundance (>9%) in the rhizosphere throughout the growth stages of *G. elata*, indicating that it might be a core fungal species under greenhouse conditions. A high relative abundance of *Mortierella* has also been observed in the fungal communities of *G. elata* by Wang et al. (2023) and Yu et al. (2023). *Mortierella* species in agricultural soils, the rhizosphere, and vegetable tissues are plant growth-promoting fungi (Ozimek and Hanaka, 2020). They increase bioavailable P, form Fe complexes, and produce phytoregulators, such as gibberellic acid, indoleacetic acid, abscisic acid, and ACC deaminase (Li et al., 2018; Ozimek et al., 2018; Zhang et al., 2020). Collectively, these results suggest that *Mortierella* species, including *M. rishikesha* are essential for improving the quality and yield of *G. elata* grown under greenhouse conditions.

The specific fungi *Mycena* spp. are required for *G. elata* seeds to germinate and develop into protocorms and subsequently switch to *A. mellea* late in protocorm development to yield mature tubers (Xu and Mu, 1990; Park et al., 2012). This replacement provides *G. elata* with access to the larger organic carbon reserves contained in the massive woody substrates metabolized by *A. mellea* (Hong et al., 2002; Kim et al., 2006; Sekizaki et al., 2008). We analyzed the antagonistic activities of its tubers as well as its symbiotic fungi MJ-1 and Am52941 against soil-inhabiting fungal and bacterial strains in this study to understand the underlying mode for the changes of rhizosphere microbial communities during the growth of *G. elata*. Regarding antibacterial activity, the survival rate of *P. putida* JBC17 was not inhibited by TE, MJ-1, or Am52941, which supports the high relative abundance of *Pseudomonas* species and the increase in *P. putida* with the growth of *G. elata.* TE strongly inhibited bacterial strains such as *B. subtilis* and *V. paradoxus,* which is also consistent with their low relative abundances in the rhizosphere of *G. elata.* Am52941 and TE did not inhibit the growth of *F. graminearum*, explaining the high relative abundance of *Fusarium* genus at all growth stages of *G. elata*. TE significantly inhibited the growth of fungal strains, such as *C. novozelandicum, B. cinerea*, and *P. digitatum*, which is consistent with the decline in the relative abundance of the *Chaetomium* and *Penicillium* genera and the absence of *Botrytis* genus in the rhizosphere. Several previous studies have reported that the bacterial and fungal communities in the cultivated soil of *G. elata* were significantly altered by *Armillaria* strains (Collins et al., 2013; Wang et al., 2023) and *Mycena* strains (Yu et al., 2023). *Armillaria mellea* produces many antibacterial and antifungal compounds, such as sesquiterpene aryl esters and armillaric acid, which exhibit high inhibitory activity against Gram-positive bacteria, yeast, *Streptococcus* spp., *Mucor* spp., *Trichoderma* spp., *Rhizopus stolonifer, Fusarium* spp., and *Gliocladium viren* (Richard, 1971; Obuchi et al., 1990). *Mycena* spp. produce the biologically active triterpenoid favolon B, which inhibits the growth of *B. cinerea*, *M. miehei*, *P. variotii*, and *Penicillium notatum* (Aqueveque et al., 2005). Furthermore, *G. elata* generates antifungal proteins such as gastrodianin or gatrodia antifungal protein (GAP), which inhibit the growth of *Trichoderma virde*, *Fusarium exospores*, and *Pyricularia oryzae* (Hu and Huang, 1994), and *B. cinerea*, *Ganoderma lucidum*, *Gibberella zeae*, *Rhizoctonia solani,* and *Valsa ambiens* (Xu et al., 1998). The antibacterial and antifungal activities of TE and Am52941 indicated that *G. elata* tubers and their symbiotic fungi contributed greatly to the selection of microbial components in the rhizosphere. However, the mechanism and effect on the growth of *G. elata* require further study.

*Mycena* species, such as *Mycena chlorophos* (Liu et al., 2015) and *M. osmundicola* (Xu and Guo, 1989), play important roles in the early stages of development of *Gastrodia* species. In the present study, the relative abundance of *Mycena* spp. in the rhizosphere was very low across the growth stages of *G. elata*. A low relative abundance of *Mycena* species was also observed in the surrounding soil of *G. flavilabella* (0.3%–2.5%; Liu et al., 2015) and in all growth stages of *G. elata* in the wild (<1.0%; Chen et al., 2019). *Armillaria* was not detected in this study. Similar results have been reported in previous studies that used high-throughput sequencing to study the fungal communities of tubers and surrounding soils of *Gastrodia* species (Liu et al., 2015; Chen et al., 2019). Presumably, *Armillaria* presented as rhizomorphs along the tubers of *G. elata* and were removed during sample processing, leading to absence or very low occurrence in the rhizosphere soil. Flynn and Niehaus (1997) reported that many DNA isolation methods are unsatisfactory for black-pigmented and slow-growing Basidiomycetes such as *Armillaria* spp. However, further studies are required to have a conclusive understanding. To the best of our knowledge, two anaerobic bacterial strains, *Paracoccus endophyticus* sp. nov. (Zhang et al., 2019) and *Cellulomonas endophytica* sp. nov. (Li et al., 2020) have been isolated from the tubers of *G. elata*. In this study, we attempted to isolate endophytic bacteria and fungi several times using different procedures with various media, but no microbes were isolated from *G. elata* tubers. Our antagonistic assays showed that TE suppresses the growth of many fungal and bacterial strains, which may explain the rarity of culturable endophytic bacterial and fungal strains.

## 5 Conclusion

This study revealed that during the growth of *G. elata* under greenhouse conditions, the richness and diversity of bacterial and fungal communities decreased with the growth of *G. elata,* which was tentatively induced by the selection of *G. elata* tubers and its symbiotic fungal strains. The dominant bacteria and fungi that were potentially beneficial for improving the quality and yield of *G. elata* increased during its growth. The genus *Mortierella*, including *Mortierella rishikesha*, was especially abundant in the rhizosphere of *G. elata* grown under greenhouse conditions. To the best of our knowledge, the present study is the first report on both bacterial and fungal community structures associated with *G. elata*, providing deep insight into the dynamics of microbial communities during *G. elata* growth under greenhouse conditions. The mechanism by which *G. elata* and its symbiotic fungi reduce the microbial community composition in the rhizosphere requires further study. The contribution of each bacterial and fungal strain or their consortia to the growth of *G. elata* will be studied in the near future.

## Data availability statement

The datasets presented in this study can be found in online repositories. The names of the repository/repositories and accession number (s) can be found at: https://www.ncbi.nlm.nih.gov/, PRJNA1074006 and https://www.ncbi.nlm.nih.gov/, PRJNA1074502.

## Author contributions

NK: Formal analysis, Investigation, Methodology, Software, Writing – original draft. SD: Formal analysis, Writing – review & editing, Methodology, Software. C-SK: Investigation, Methodology, Writing – review & editing, Resources. YL: Conceptualization, Formal analysis, Funding acquisition, Investigation, Resources, Supervision, Validation, Visualization, Writing – original draft, Writing – review & editing.

## Footnotes

^1^ https://www.ezbiocloud.net/

^2^ https://unite.ut.ee
