# Supplementary material for: Features of bacterial and fungal communities in the rhizosphere of Gastrodia elata cultivated in greenhouse for early harvest
Source: Front Microbiol. 2024 Apr 24;15:1389907. doi: 10.3389/fmicb.2024.1389907 (PMC11076825; doi:10.3389/fmicb.2024.1389907)
Supplement: Supplementary file 1 [file Data_Sheet_1.docx]

Supplementary Information

# . Supplementary Figures and Tables

## Supplementary Figures


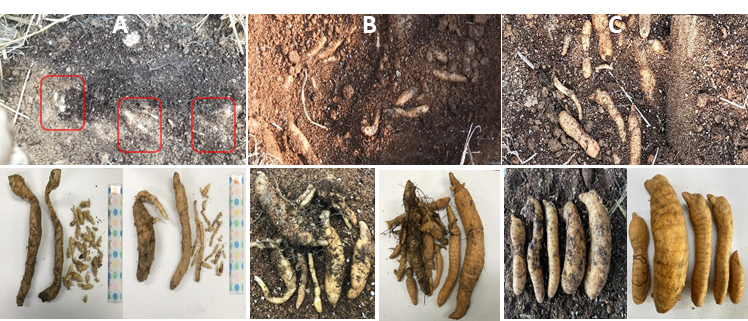


Supplementary Figure S1. Samples of *Gastrodia elata* tubers and its associated soils that are cultivated in greenhouse. *G. elata* tubers and surrounding soils were collected from the greenhouse at (A) rooting and juvenile tuber (JT, 2 months after sowing; MAS), (B) growing and young tuber (YT, 6 MAS), and then (C) harvesting and mature tuber (MT, 12 MAS) stages.


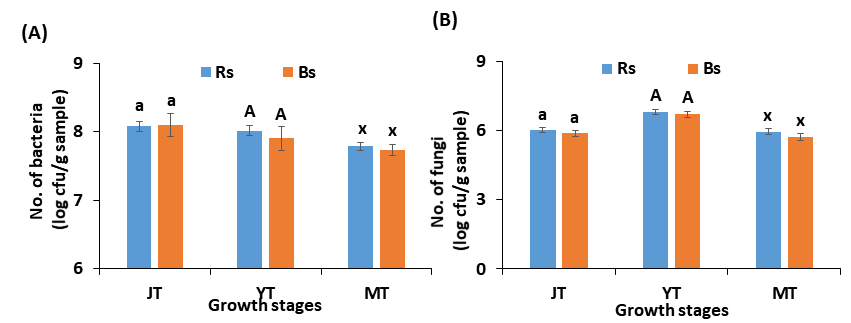


Supplementary Figure S2. Total number of culturable bacteria and fungi in the soils surrounding tubers of *G. elata*. (A) Bacterial and (B) fungal numbers were estimated from bulk soil (Bs) and rhizosphere soil (Rs) of greenhouse at various growing stages of *G. elata;* rooting and juvenile tuber (JT, 2 months after sowing; MAS), growing and young tuber (YT, 6 MAS), and harvesting and mature tuber (MT, 12 MAS) stages. Data presented as mean±SD and bars with same letter do not differ significantly (ANOVA, p>0.05).


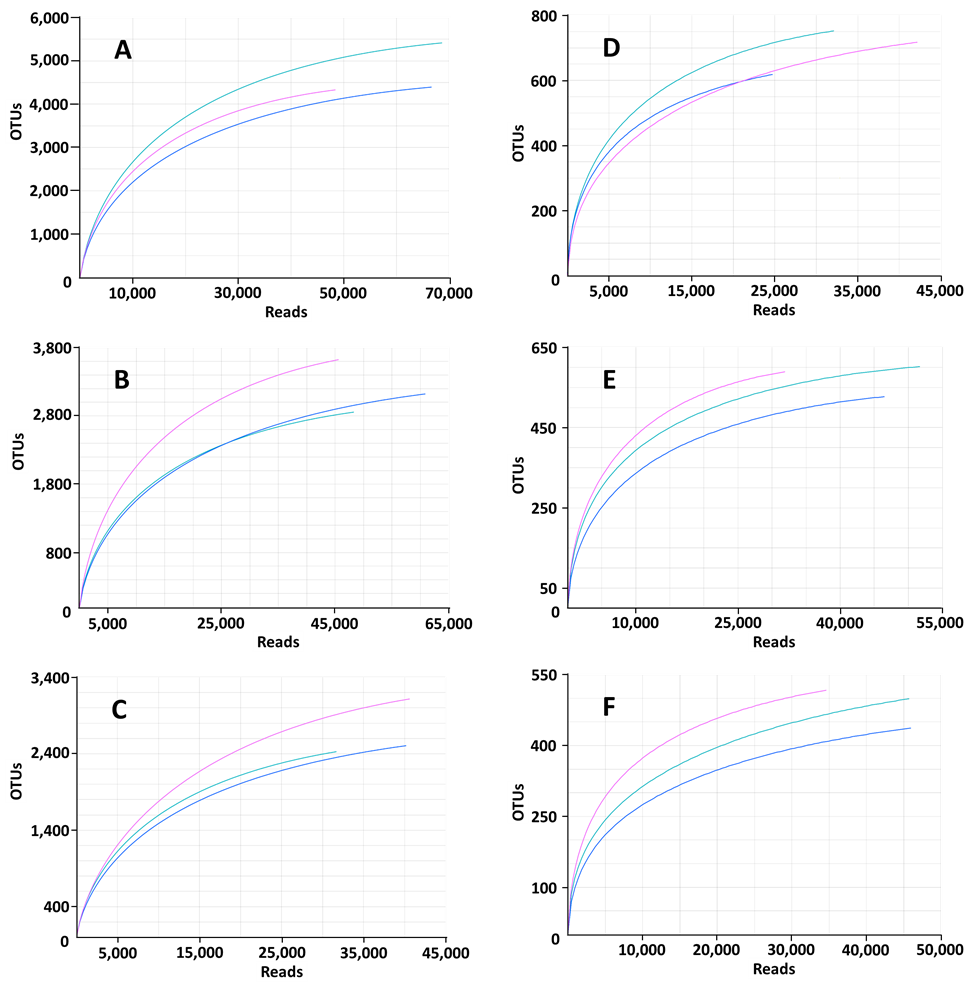


Supplementary Figure S3. Rarefaction curves for bacterial and fungal operational taxonomic units (OTUs) of *Gastrodia elata* rhizosphere. Rhizosphere soil of *G. elata* at rooting and juvenile tuber (A and D, left and right column indicates bacteria and fungi, respectively), growing and young tubers (B and E), and harvesting and mature tuber (C and F) stages were collected from greenhouse and analyzed with cut-off value at 97% similarity. The vertical axis indicates the number of OTUs expected after sampling the number of sequences denoted in the horizontal axis.

**
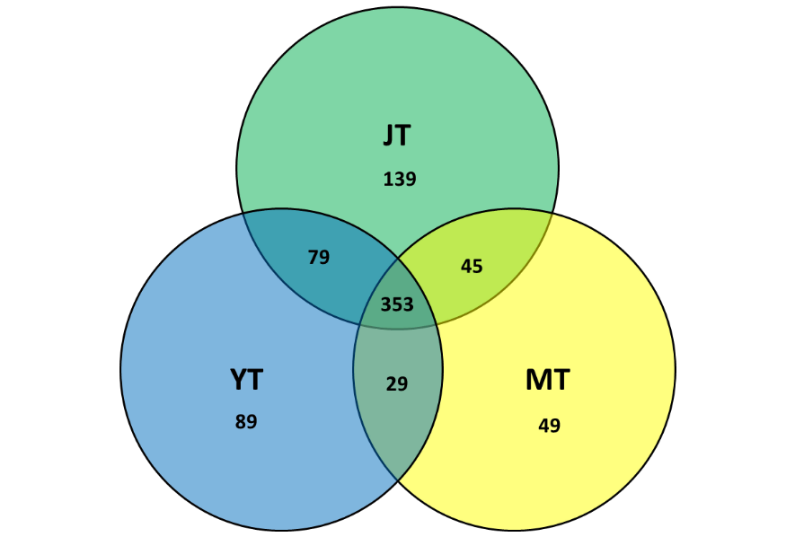
**

Supplementary Figure S4. Venn diagram for bacterial communities at genera. The number of bacterial genera in each growth stage. The bacterial genera with more than 0.01% relative abundance were assessed from rhizosphere soil at different development stages, juvenile tuber (JT), young tuber (YT) and mature tuber (MT) stages of *G. elata* under greenhouse condition.


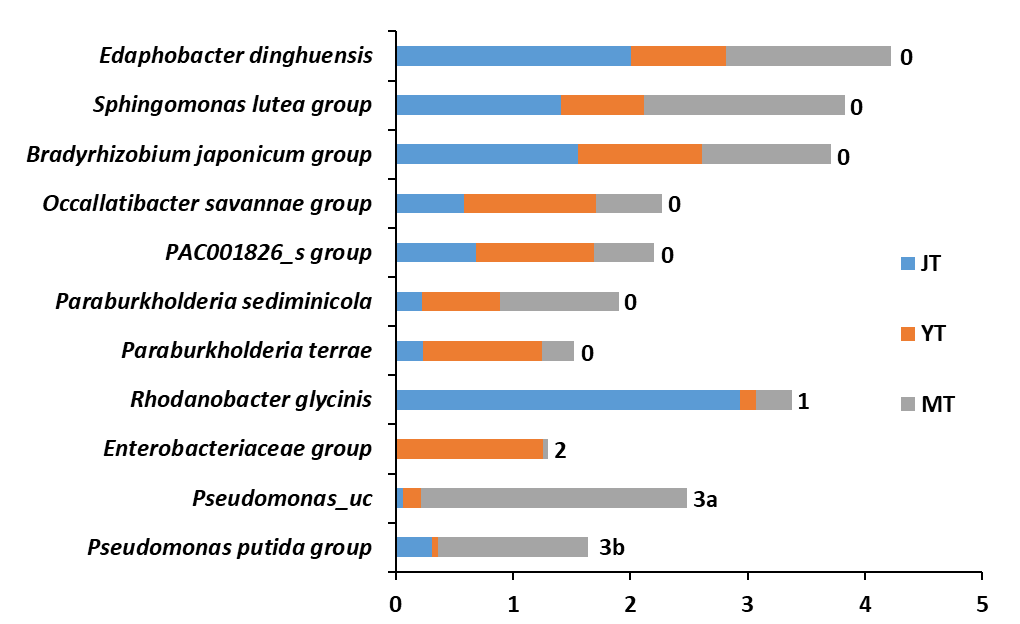


Supplementary Figure S5. Relative abundance of bacterial species in rhizosphere soil of *Gastrodia elata*. The bacterial species with relative abundances more than 1.0 % in rhizosphere soil of greenhouse at juvenile tuber (JT), young tuber (YT), and mature tuber (MT) stages were compared. The number in right side of each column indicate (0) JT=YT=MT; (1) JT>YT=MT; (2) YT>JT=MT; (3a) MT>YT=JT; (3b) MT≥JT≥YT (ANOVA, p<0.05).


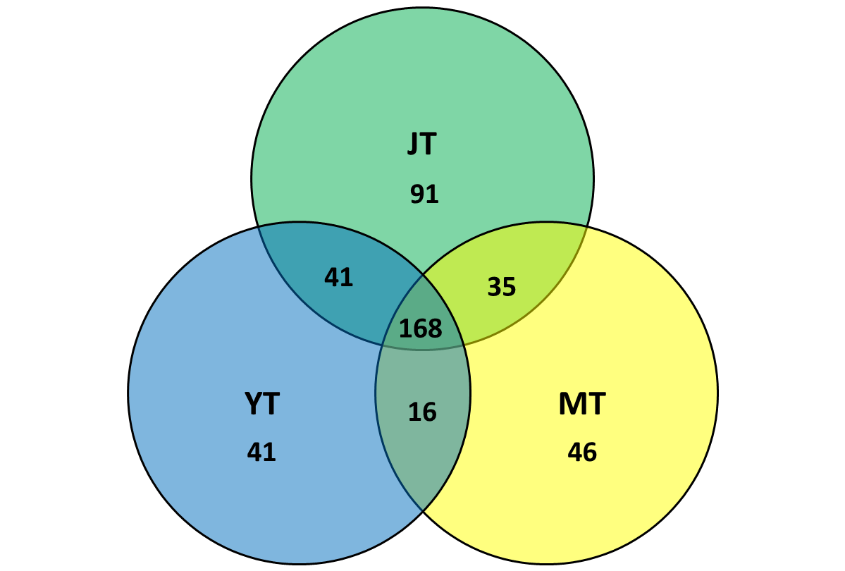


Supplementary Figure S6. Venn diagram for fungal communities at genera. The fungal genera were assessed from rhizosphere soil at different development stages, juvenile tuber (JT), young tuber (YT), and mature tuber (MT) stages of *G. elata* under greenhouse condition.


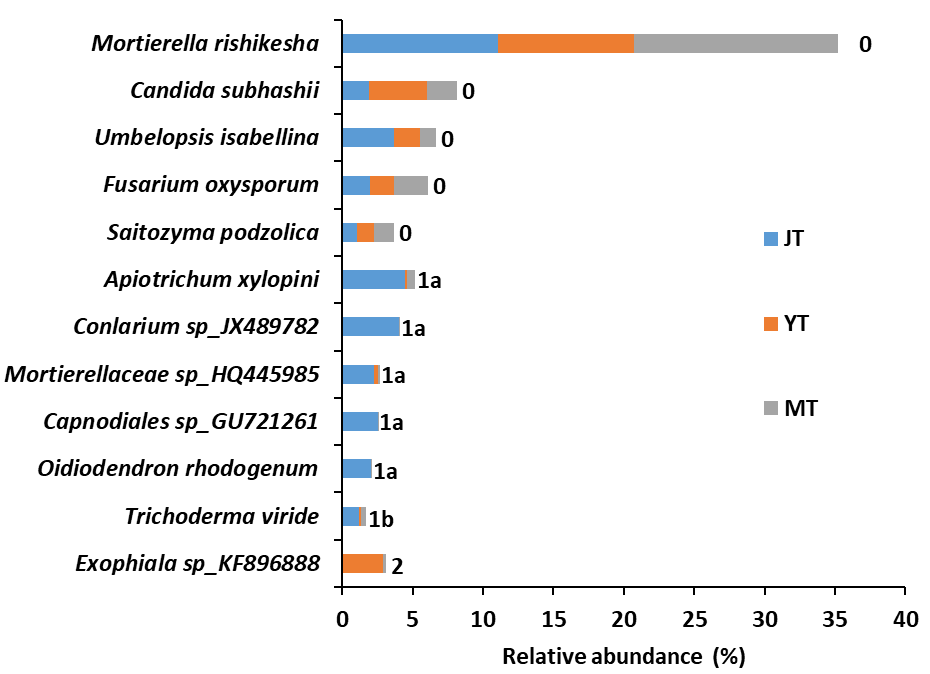


Supplementary Figure S7. Relative abundance of fungal species in rhizosphere soil of *Gastrodia elata.* The fungal species with relative abundances more than 1.0 % in rhizosphere soil of greenhouse at juvenile tuber (JT), young tuber (YT), and mature tuber (MT) stages were compared. The numbers in right side of each column indicate (0) JT=YT=MT; (1a) JT>YT=MT; (1b) JT≥MT≥YT; (2) YT≥MT≥JT (ANOVA, p<0.05).


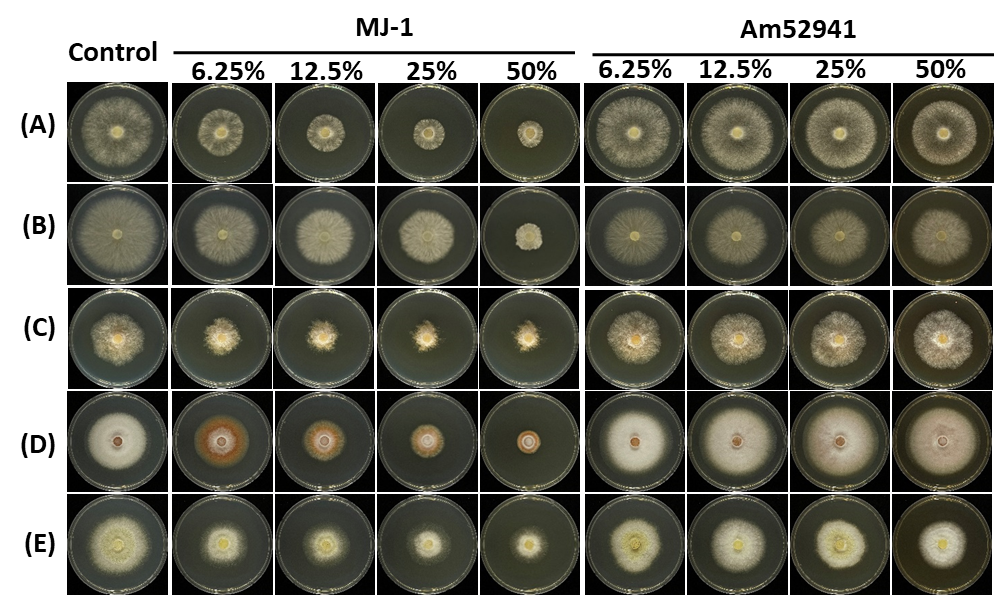


Supplementary Figure S8. Inhibition of mycelial growth of fungi by various concentration of culture filtrate of *Mycena* sp. and *Armillaria mellea*. PDA mixed with each concentration (6.25 %, 12.5 %, 25 %, and 50 %) of culture filtrated supernatant of *Mycena* sp. Jinan-1 (MJ-1) and *Armillaria mellea* KACC52941 (Am52941) was poured on the plates, and then each mycelial plug, (A) *Botrytis cinerea*; (B) *Chaetomium novozelandicum;* (C) *Fusarium graminearum* KACC41040*;* (D) *Penicillium digitatum*; (E) *Rhizopus stolonifer* KACC41364 was inoculated in the center of the PDA plates.


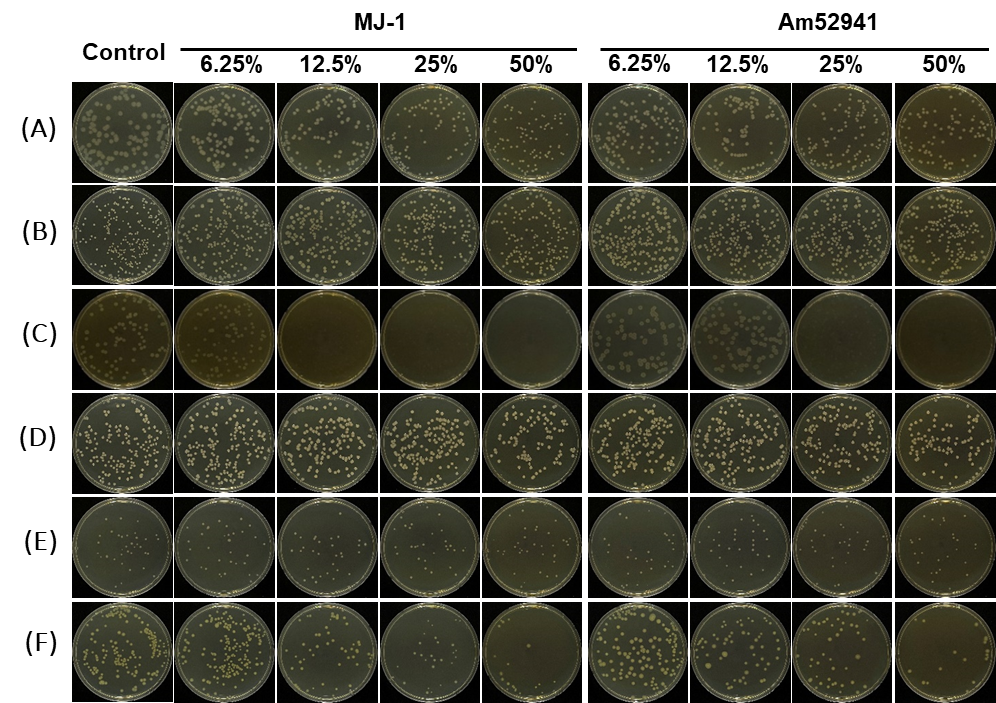


Supplementary Figure S9. Inhibition of bacterial cell survival by various concentration of culture filtrate of *Mycena* sp. Jinan-1(MJ-1) and *Armillaria mellea* KACC52941 (Am52941). LB media mixed with each concentration (6.25 %, 12.5 %, 25 %, and 50 %) of culture filtrated supernatant was poured on the plates and bacterial cell suspension of (A) *Bacillus subtilis;* (B) *Bacillus velezensis;*(C) *Cytobacillus firmus;* (D) *Agrobacterium tumefaciens (E) Pseudomonas putida; (F) Variovorax paradoxus* was spread on the plates.

**
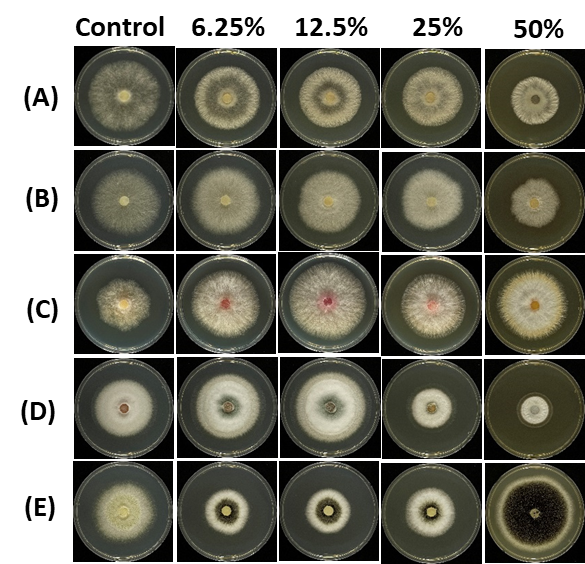
**

Supplementary Figure S10. Inhibition of mycelial growth of fungi by the addition of various concentration of tuber extract of *G. elata*. PDA mixed with each concentration (6.25 %, 12.5 %, 25 %, and 50 %) of tuber extract was poured on the plates and each mycelial plug, (A) *Botrytis cinerea*; (B) *Chaetomium novozelandicum;* (C) *Fusarium graminearum* KACC41040*;* (D) *Penicillium digitatum*; (E) *Rhizopus stolonifer* KACC41364 was inoculated in the center of the PDA plates.

*
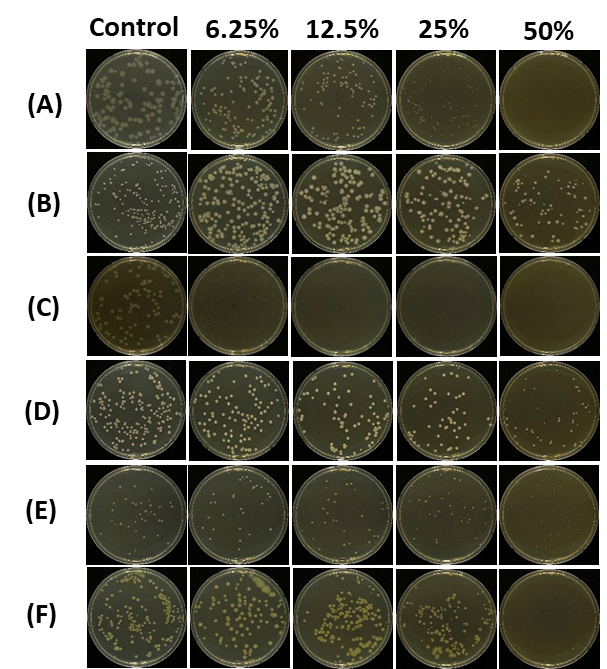
*

Supplementary Figure S11. Inhibition of bacterial cell survival by various concentration of tuber extract of *G. elata*. LB mixed with each concentration (6.25 %, 12.5 %, 25 %, and 50 %) of culture filtrated supernatant was poured on the plates and bacterial cell suspension of (A) *Bacillus subtilis;* (B) *Bacillus velezensis;* (C) *Cytobacillus firmus;* (D) *Agrobacterium tumefaciens* (E) *Pseudomonas putida;* (F) *Variovorax paradoxus* was spread on the plates.

## Supplementary Tables

Supplementary Table S1. Estimated bacterial OTU richness and diversity indices in rhizosphere of *Gastrodia elata* in greenhouse

| Sample Name | | Valid  reads | | OTUs | | ACE | Chao1 | Jackknife | | NPShannon | | Shannon | | Pielou’s evenness | | Simpson | | Phylogenetic Diversity | | Good's coverage of library (%) | |  |
| --- | --- | --- | --- | --- | --- | --- | --- | --- | --- | --- | --- | --- | --- | --- | --- | --- | --- | --- | --- | --- | --- | --- |
| JT | 1 | 68637 | 5416 | | 5867.03 | | 5648.17 | | 6210 | | 7.26 | | 7.17 | | 0.87 | | 0.00 | | 5597 | | 98.84 | |
|  | 2 | 66667 | 4394 | | 4835.08 | | 4643.29 | | 5117 | | 6.87 | | 6.79 | | 0.84 | | 0.00 | | 4819 | | 98.92 | |
|  | 3 | 48426 | 4330 | | 4782.67 | | 4582.62 | | 5076 | | 7.05 | | 6.95 | | 0.86 | | 0.00 | | 4969 | | 98.46 | |
| YT | 1 | 48297 | 2856 | | 3242.74 | | 3095.95 | | 3421 | | 6.08 | | 6.01 | | 0.78 | | 0.01 | | 3365 | | 98.83 | |
|  | 2 | 60890 | 3122 | | 3571.03 | | 3402.39 | | 3761 | | 5.94 | | 5.87 | | 0.75 | | 0.01 | | 3877 | | 98.95 | |
|  | 3 | 45608 | 3624 | | 4122.49 | | 3913.85 | | 4354 | | 6.61 | | 6.51 | | 0.82 | | 0.01 | | 4490 | | 98.40 | |
| MT | 1 | 31701 | 2427 | | 2844.20 | | 2690.29 | | 2991 | | 6.00 | | 5.90 | | 0.78 | | 0.01 | | 3194 | | 98.22 | |
|  | 2 | 40202 | 2505 | | 2949.77 | | 2790.15 | | 3089 | | 5.86 | | 5.77 | | 0.76 | | 0.01 | | 3430 | | 98.55 | |
|  | 3 | 40631 | 3116 | | 3636.01 | | 3429.97 | | 3838 | | 6.06 | | 5.96 | | 0.76 | | 0.01 | | 4002 | | 98.22 | |

Supplementary Table S2. Estimated fungal OTU richness and diversity indices in rhizosphere of *Gastrodia elata* in greenhouse

| Sample Name | | Valid  reads | OTUs | ACE | Chao1 | Jackknife | NPShannon | Shannon | Pielou’s evenness | Simpson | Phylogenetic Diversity | Good's coverage of library (%) |
| --- | --- | --- | --- | --- | --- | --- | --- | --- | --- | --- | --- | --- |
| JT | 1 | 32127 | 752 | 830.25 | 800.24 | 874 | 4.34 | 4.31 | 0.68 | 0.06 | 1099 | 99.62 |
|  | 2 | 24742 | 618 | 705.80 | 683.90 | 740 | 4.61 | 4.58 | 0.74 | 0.02 | 958 | 99.51 |
|  | 3 | 42172 | 717 | 814.05 | 782.69 | 856 | 4.04 | 4.01 | 0.64 | 0.05 | 1069 | 99.67 |
| YT | 1 | 51719 | 602 | 643.64 | 624.66 | 675 | 3.55 | 3.53 | 0.60 | 0.09 | 766 | 99.86 |
|  | 2 | 46517 | 527 | 576.94 | 552.89 | 605 | 3.03 | 3.02 | 0.51 | 0.15 | 774 | 99.83 |
|  | 3 | 31907 | 589 | 640.36 | 618.48 | 675 | 3.82 | 3.80 | 0.65 | 0.07 | 782 | 99.73 |
| MT | 1 | 45742 | 499 | 597.88 | 567.31 | 611 | 3.43 | 3.41 | 0.56 | 0.12 | 863 | 99.76 |
|  | 2 | 45974 | 437 | 514.78 | 493.77 | 531 | 3.27 | 3.25 | 0.55 | 0.09 | 766 | 99.80 |
|  | 3 | 34636 | 517 | 586.13 | 567.33 | 610 | 3.99 | 3.97 | 0.65 | 0.04 | 840 | 99.73 |

Supplementary Table S3. Comparison of relative abundance of bacterial composition at phylum level with relative abundances more than 1%

| Phylum | Relative abundance (%)^*^ | | |
| --- | --- | --- | --- |
|  | JT | YT | MT |
| Acidobacteria | 24.61± 1.60^a^ | 23.77 ± 5.49^a^ | 21.15 ± 2.21^a^ |
| Actinobacteria | 8.62 ± 0.72^a^ | 3.31 ± 0.86^a^ | 8.15 ± 3.73^a^ |
| Bacteroidetes | 6.10 ± 0.76^a^ | 12.80 ± 7.12^a^ | 13.21 ± 3.90^a^ |
| Chloroflexi | 1.71 ± 0.13^a^ | 0.96 ± 0.60^a^ | 2.20 ± 0.60^a^ |
| Gemmatimonadetes | 1.80 ± 0.30^a^ | 0.90 ± 0.22^b^ | 1.03 ± 0.33^b^ |
| Parcubacteria_OD1 | 1.20 ± 0.62^a^ | 1.21 ± 0.49^a^ | 1.35 ± 0.15^a^ |
| Planctomycetes | 1.93 ± 0.25^a^ | 1.62 ± 0.22^ab^ | 1.34 ± 0.23^b^ |
| Proteobacteria | 40.61 ± 0.89^a^ | 45.17 ± 3.11^a^ | 43.03 ± 0.75^a^ |
| Saccharibacteria_TM7 | 2.85 ± 0.66^a^ | 0.84 ± 0.08^b^ | 1.22 ± 0.12^b^ |
| Verrucomicrobia | 6.96 ± 1.75^a^ | 5.90 ± 1.58^a^ | 4.49 ± 1.35^a^ |

^*^The data are the mean relative abundances (mean±SD) of three replicates and significant differences are presented by different letters in the same row (ANOVA, p<0.05).

Supplementary Table S4. Comparison of relative abundance of bacterial composition at genus level with relative abundances more than 1%.

| Phylum | Genus | Relative abundance (%)^*^ | | |
| --- | --- | --- | --- | --- |
|  |  | JT | YT | MT |
| Acidobacteria | *Edaphobacter* | 2.47 ± 0.27^a^ | 2.98 ± 2.58^a^ | 1.58 ± 0.94^a^ |
|  | *Occallatibacter* | 1.11 ± 0.27^a^ | 2.60 ± 1.55^a^ | 0.65 ± 0.41^a^ |
|  | *Solibacter* | 1.34 ± 0.15^ab^ | 1.81 ± 0.31^a^ | 0.72 ± 0.37^b^ |
| Actinobacteria | *Arthrobacter* | 1.31 ± 0.51^a^ | 0.10 ± 0.05^a^ | 1.39 ± 0.91^a^ |
|  | *Kitasatospora* | 1.09 ± 0.79^a^ | 0.05 ± 0.03^a^ | 0.57 ± 0.27^a^ |
| Bacteroidetes | *Chryseobacterium* | 0.17 ± 0.11^a^ | 1.31 ± 1.16^a^ | 1.07 ± 0.53^a^ |
|  | *Flavobacterium* | 0.25 ± 0.08^a^ | 2.64 ± 2.26 ^a^ | 6.58 ± 4.28^a^ |
|  | *Mucilaginibacter* | 2.88 ± 0.82^a^ | 1.72 ± 1.26^a^ | 3.33 ± 1.36^a^ |
| Proteobacteria | *Acidibacter* | 0.76 ± 0.07^ab^ | 1.27 ± 0.59^a^ | 0.26 ± 0.05^b^ |
|  | *Bradyrhizobium* | 0.90 ± 0.01^ab^ | 1.08 ± 0.18^a^ | 0.47 ± 0.30^b^ |
|  | *Pseudolabrys* | 0.62 ± 0.04^b^ | 1.20 ± 0.28^a^ | 0.51 ± 0.05^b^ |
|  | *Pseudomonas* | 1.95 ± 1.09^b^ | 1.32 ± 1.08^b^ | 15.19 ± 5.00^a^ |
|  | *Rahnella* | 0.02 ± 0.01^b^ | 0.21 ± 0.18^b^ | 2.96 ± 1.54^a^ |
|  | *Rhizobium* | 1.33 ± 0.28^a^ | 1.46 ± 1.04^a^ | 2.14 ± 1.04^a^ |
|  | *Rhizomicrobium* | 4.24 ± 0.24^a^ | 4.44 ± 1.77^a^ | 2.35 ± 0.60^a^ |
|  | *Rhodanobacter* | 5.01 ± 0.82^a^ | 0.37 ± 0.24^b^ | 1.05 ± 0.73^b^ |
|  | *Sphingomonas* | 3.99 ± 1.01^a^ | 1.73 ± 0.26^a^ | 3.62 ± 2.88^a^ |
| Verrucomicrobia | *Limisphaera* | 1.86 ± 0.74^a^ | 1.50 ± 0.41^a^ | 0.85 ± 0.50^a^ |

^*^The data are the mean relative abundances (mean±SD) of three replicates and significant differences are presented by different letters in the same row (ANOVA, p<0.05).

Supplementary Table S5. Comparison of relative abundance of fungal composition at phylum level with relative abundances more than 1%.

| Phylum | Relative abundance (%)^*^ | | |
| --- | --- | --- | --- |
|  | JT | YT | MT |
| Ascomycota | 48.32 ± 7.23^ab^ | 22.23 ± 7.37^b^ | 49.48 ± 14.93^a^ |
| Basidiomycota | 21.87 ± 9.65^a^ | 33.10 ± 23.90^a^ | 19.90 ± 6.90^a^ |
| Mortierellomycota | 18.31 ± 8.57^a^ | 13.59 ± 5.04^a^ | 19.30 ± 16.27^a^ |
| Mucoromycota | 6.70 ± 1.31^a^ | 4.71 ± 1.59^a^ | 5.74 ± 4.00^a^ |
| Fungi_p | 2.25 ± 0.16^a^ | 24.50 ± 20.60^a^ | 5.20 ± 6.84^a^ |

^*^The data are the mean relative abundances (mean±SD) of three replicates and significant differences are presented by different letters in the same row (ANOVA, p<0.05).

Supplementary Table S6. Comparison of relative abundance of fungal composition at genus level with relative abundances more than 1%.

| Phylum | Genus | Relative abundance (%)^*^ | | |
| --- | --- | --- | --- | --- |
|  |  | JT | YT | MT |
| Ascomycota | *Ascomycota_g* | 0.70 ± 0.44^a^ | 0.47 ± 0.27^a^ | 1.21 ± 0.86^a^ |
|  | *Candida* | 1.97 ± 0.70^a^ | 4.26 ± 1.00^a^ | 2.16 ± 1.10^a^ |
|  | *Capnodiales_g* | 2.52 ± 0.53^a^ | 0.02 ± 0.0^b^ | 0.06 ± 0.02^b^ |
|  | *Chaetomium* | 9.03 ± 4.80^a^ | 1.43 ± 1.07^a^ | 5.76 ± 8.71^a^ |
|  | *Chrysosporium* | 1.22 ± 0.98^a^ | 0.12 ± 0.02^a^ | 0.22 ± 0.14^a^ |
|  | *Conlarium* | 5.03 ± 2.89^a^ | 0.06 ± 0.02^b^ | 0.02 ± 0.01^b^ |
|  | *Exophiala* | 0.47 ± 0.21^a^ | 3.19 ± 2.15^a^ | 0.53 ± 0.12^a^ |
|  | *Fusarium* | 2.54 ± 0.25^a^ | 1.90 ± 0.83^a^ | 2.79 ± 2.22^a^ |
|  | *Oidiodendron* | 2.46 ± 0.62^a^ | 0.06 ± 0.03^b^ | 0.11 ± 0.07^b^ |
|  | *Pseudogymnoascus* | 1.72 ± 0.77^a^ | 0.33 ± 0.13^a^ | 3.30 ± 2.51^a^ |
|  | *Sordariomycetes_g* | 1.69 ± 0.39^a^ | 0.33 ± 0.19^b^ | 0.42 ± 0.30^b^ |
|  | *Trichoderma* | 3.30 ± 2.07^a^ | 1.04 ± 0.66^a^ | 1.64 ± 1.22^a^ |
| Basidiomycota | *Apiotrichum* | 4.44 ± 2.55^a^ | 0.14 ± 0.08^b^ | 0.60 ± 0.73^b^ |
|  | *Auriculariales_g* | 0.03 ± 0.06^b^ | 2.19 ± 1.18^a^ | 0.24 ± 0.15^b^ |
|  | *Saitozyma* | 1.03 ± 0.22^a^ | 1.22 ± 0.50^a^ | 1.44 ± 1.11^a^ |
|  | *Solicoccozyma* | 0.87 ± 0.64^a^ | 0.64 ± 0.25^a^ | 2.41 ± 1.64^a^ |
| Mortierellomycota | *Mortierella* | 15.55 ± 8.99^a^ | 13.17 ± 4.95^a^ | 19.09 ± 16.23^a^ |
|  | *Mortierellaceae_g* | 2.42 ± 0.87^a^ | 0.33 ± 0.23^b^ | 0.18 ± 0.08^b^ |
| Mucoromycota | *Umbelopsis* | 4.14 ± 0.97^a^ | 1.97 ± 1.33^ab^ | 1.27 ± 0.83^b^ |

^*^ The data are the mean relative abundances (mean±SD) of three replicates and significant differences are presented by different letters in the same row (ANOVA, p<0.05).

Supplementary Table S7. Radius of mycelium of fungal strains that co-cultured with *Mycena* sp. Jinan-1 (MJ-1) or *Armillaria mellea* KACC52941 (Am52941) on PDA plates.

| Fungal strains | Radius of mycelium (mm)^*^ | | Control |
| --- | --- | --- | --- |
|  | MJ-1 | Am52941 |  |
| *Botrytis cinerea* | 23.5 ± 1.0^c^  (55.4) ^**^ | 34.3 ± 1.9^b^  (34.9) | 52.8 ± 3.5^a^ |
| *Chaetomium novozelandicum* | 25.3 ± 0.9^c^  (48.9) | 41.2 ± 2.3^b^  (16.7) | 49.5 ± 2.0^a^ |
| *Fusarium graminearum* | 29.4 ± 1.8^b^  (39.4) | 50.3 ± 1.9^a^  (NI) | 48.5 ± 2.3^a^ |
| *Penicillium digitatum* | 17.9 ± 0.9^c^  (46.1) | 25.4 ± 1.2^b^  (23.4) | 33.2 ± 2.1^a^ |
| *Rhizopus stolonifer* | 21.1 ± 1.6^c^  (54.7) | 28.2 ± 0.8^b^  (39.5) | 46.6 ± 1.8^a^ |

^*^The data are the mean radius of mycelia (mean±SD) of three replicates and significant differences are presented by different letters in the same row (ANOVA, p<0.05).^**^ Inhibition rate (%), NI, no inhibition.
